# Enhancement of river flooding due to global warming

**DOI:** 10.1038/s41598-022-25182-6

**Published:** 2022-11-30

**Authors:** Haireti Alifu, Yukiko Hirabayashi, Yukiko Imada, Hideo Shiogama

**Affiliations:** 1 Department of Civil Engineering, Shibaura Institute of Technology, Tokyo, Japan; 2 Taylor Geospatial Institute, Saint Louis University, St. Louis, USA; 3 Meteorological Research Institute, Tsukuba, Japan; 4 National Institute for Environmental Studies, Tsukuba, Japan

**Keywords:** Hydrology, Atmospheric science, Climate change

## Abstract

Human-induced climate change has increased the frequency and intensity of heavy precipitation^1^. Due to the complexity of runoff generation and the streamflow process, the historical impact of human-induced climate change on river flooding remains uncertain. Here, we address the question of whether anthropogenic climate change has altered the probability of the extreme river flood events for the period 1951–2010 based on simulated river discharge derived from large ensemble climate experiments with and without human-induced climate change. The results indicate that human-induced climate change altered the probabilities of 20 of the 52 analyzed flood events. Fourteen of these 20 flood events, which occurred mainly in Asia and South America, were very likely to have been enhanced by human-induced climate change due to an increase in heavy precipitation. Conversely, two flood events in North/South America and two flood events in Asia and two flood events in Europe were suppressed by human-induced climate change, perhaps as a result of lower snowfall. Human-induced climate change has enhanced flooding more prominently in recent years, providing important insights into potential adaptation strategies for river flooding.

## Introduction

Extreme weather events (e.g., heavy precipitation and flooding) can have devastating effects on human society and the environment. Heavy precipitation events, a major contributor to flooding, have increased in recent years, especially in many parts of the Northern Hemisphere, due to human-induced climate change (increases in greenhouse gases, GHGs)^1–4^. Despite the fact that floods are projected to occur frequently in many regions as the climate warms further^5^, the effects of past human-induced climate change on river flooding remain unclear due to the complexity of runoff generation and streamflow processes.

The latest 6th Intergovernmental Panel on Climate Change (IPCC) Assessment Report stated that there is low confidence regarding the changes in past floods on a global scale due to human-induced climate change because of limited evidence^5,6^. One reason for this is that there are spatial and temporal limitations in the available observed data (especially from data-poor regions such as Asia and Africa), as well as in the tools used to gather such data^7^. Fortunately, in recent years it has become possible to investigate historical flooding using newly developed models, such as the global river inundation model, which can replicate changes in long-term river flow, and large ensemble climate experiments. A study based on these tools found that the spatial patterns of observed trends in river flooding can be reproduced only when human-induced climate change is considered^8^. Another recent advancement in attributing past flooding changes is event attribution (EA) using the large ensemble climate experiments derived from Atmospheric General Circulation Models (AGCMs), which is a method of probabilistically evaluating the occurrence of extreme phenomena over a specific period in which extreme phenomena are observed^9^. These climate models have the advantage of being able to represent most important atmosphere and land processes using specific input conditions (e.g., sea-surface temperature [SST], sea ice cover, and human-induced climate changes including concentrations of atmospheric CO_2_ and aerosols). Moreover, the models are able to create a factual world with climate warming (referred to as HPB, see Methods) and a hypothetical counterfactual world without climate warming (referred to as NAT). The experiments aim to reproduce the atmospheric conditions under which extreme phenomena occur by providing the same oceanic natural variability of an extreme phenomenon^10^. The probability of event occurrence can then be estimated by repeating the simulation many times (ensemble). It is possible to quantitatively show the effects of human-induced climate change on past extreme phenomena by comparing the probability of the occurrence between factual (with climate change) and hypothetical counterfactual climate simulations. Thus far, the EA has been used to measure quantitatively the contribution of human-induced climate change to past specific extreme phenomena^1,10–13^. Applying this EA method to globally distributed river flooding indicated that the probability of occurrence of 14 of the 22 flood events analyzed between 2010 and 2013 was influence by human-induced climate change^13^. Spring floods due to snowmelt are more susceptible to historical warming due to increased precipitation in the Northern Hemisphere and decreased snowfall due to increased temperatures^13^. However, we cannot clarify whether the impact of flooding has changed over time, since climate experiments are available only for a short target period. To address this limitation, we extended this study to include a larger number of flood events that occurred over a longer time period (1951–2010) using data from a large ensemble climate experiment, the “Database for Policy Decision-Making for Future Climate Change” (d4PDF)^14^, derived from the Meteorological Research Institute (MRI) AGCM 3.2 and CaMa-Flood (Catchment-based Macro-Scale Floodplain) model^15^.

**Figure 1:**
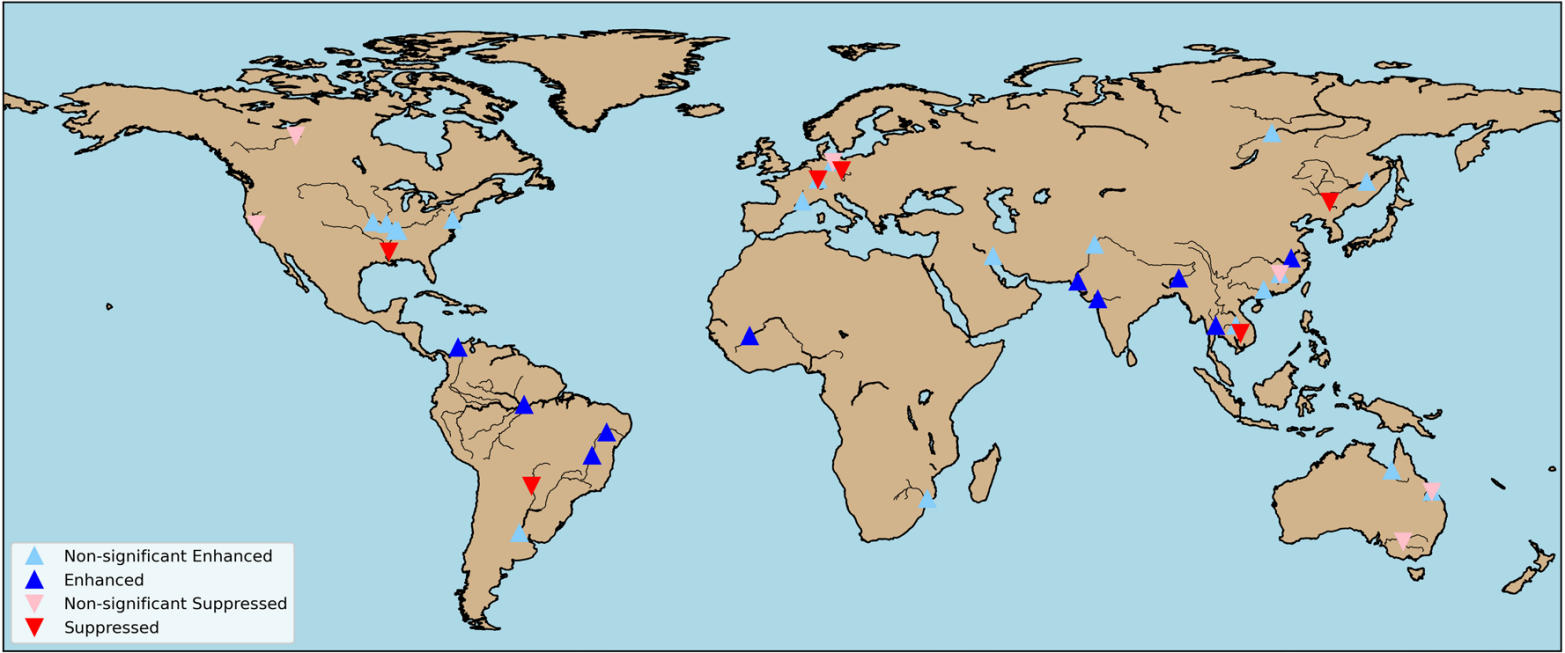
Global distribution of flood events included in this study. This figure was created using Python (3.9.7) and the Basemap library (1.2.2).

Flood events were determined from the Emergency Events Database (EM-DAT), news media, streamflow records, global daily S14FD discharge reanalysis (Supplementary Materials S1), and existing publications. Data were collected that included the year of occurrence, location, reported damage, number of victims, and main causes of flooding. For selected flood events, we then confirmed whether the flood magnitude in the year of occurrence statistically exceeded the magnitude of a 10-year flood based on available observations, S14FD discharge reanalysis, and HPB simulation. In total, 52 globally distributed flood events (Fig. 1 and Supplementary Table 1) were identified. Forty-one of the 52 flood events in 28 river basins occurred after 1980. Nearly 38% of flood events occurred in Asia, followed by South America (12 events), North America (9 events), Europe (6 events), Oceania (5 events), and Africa (3 events). The fraction of attribution risk (FAR)^16^ was used to quantify the influence of human-induced climate change on these historical events by comparing the probability of the flood events in the historical climate simulation (hereafter called as HPB) and NAT (counterfactual experiments without human-induced climate change) (see Methods). The bootstrap resampling technique was used to quantify uncertainty in the FAR by calculating the FAR 1000 times using randomly selected ensembles with replacement of all available ensemble simulations.

The results demonstrated that ongoing climate change affected the occurrence of flood events. Among the 52 selected flood events for the period 1951–2010, human-induced climate change changed the occurrence of 20 flood events with 90% likelihood; most of which occurred in Asia and South America (Fig. 1, Table 1, and Supplementary Fig. 4). The occurrence of 14 flood events in Asia (Yangtze River, Indus River, Brahmaputra River, Tapi River, and Chao Phraya River), South America (Magdalena River, Amazon River, and Sao Francisco River), and Africa (Niger River) increased. These flood events were caused mainly by heavy rainfall, including monsoon-induced heavy rainfall in Asia and rainfall associated with El Nino (Yangtze River) or La Nina (Magdalena River). Assessment of rainfall-related indices also indicated that heavy precipitation in the above-mentioned flood events occurred more frequently in HPB experiments than in NAT experiments. In particular, the quantity of short-duration heavy precipitation (annual maximum 1-day or 5-day precipitation) was greater in HPB than NAT for these 14 intensified flood events, except for one event in the Sao Francisco River in 1979. Similarly, more precipitation occurred in almost all events with insignificant but enhanced flooding (Table 1). These results were consistent with those of previous studies, suggesting that short-duration heavy precipitation has intensified, and that this trend has been significantly driven by human-induced thermodynamic changes, including increases in atmospheric moisture^1,5,6,8,17,18^.

**Table 1:** Summary of selected flood events with significant effects of human-induced climate change (E, enhanced; S, suppressed). Changes in climate variables are derived as the average of the upper basin area of the station (number of GRDC stations are given in Supplementary Table 1). The numbers correspond to the event number in the Supplementary Table S1.

	Region	River	Year	Causes	Effect		
					M5D	Median of annual mean	
1	AF	NIGER	1963	HR^14^	9.36	63.81	E*
4	AS	SONGHUA	1975	HR^17^	3.79	− 4.06	S*
7	AS	YANGTZE	1998	HRE^20^	5.91	44.94	E*
11	AS	INDUS	1988	HR^24^	9.42	− 51.85	E*
13	AS	MEKONG	1996	HRS^26^	3.30	61.89	S*
14	AS	BRAHMAPUTRA	1998	HRM^27^	11.24	21.33	E*
15	AS	TAPI	1968	HR^28^	5.35	46.30	E*
18	AS	CHAO PHRAYA	1995	HR^31^	4.53	57.42	E*
19	AS	CHAO PHRAYA	2010	HR^32^	4.98	58.06	E*
21	SA	MAGDALENA	1973	HR^34^	2.01	67.60	E*
23	SA	MAGDALENA	2008	HRL^35^	3.99	68.15	E*
24	SA	MAGDALENA	2010	HRL^35^	4.27	67.41	E*
27	SA	AMAZON	2000	HR^37^	2.90	68.64	E*
28	SA	AMAZON	2006	HR^37^	1.66	67.12	E*
29	SA	SAO FRANCISCO	1979	HR^38^	− 4.04	− 9.73	E*
31	SA	SAO FRANCISCO	1980	HR^40^	1.46	21.11	E*
32	SA	RIO PARAGUAY	1980	HR^41^	− 0.43	27.78	S*
37	NA	MISSISSIPPI	1975	HR^45^	2.51	27.72	S*
48	EU	RHINE	1983	HR^54^	3.04	58.79	S*
50	EU	ELBE	1995	HR^56^	0.67	26.42	S*

AF, Africa; AS, Asia; EU, Europe; SA, South America; NA, North America; OC, Oceania; N/A, not available. HR, Heavy rain; M, monsoon; E, El Nino; S, Strom; L, La Niña; SM, Snowmelt. M5D, annual maximum daily precipitation. Asterisks indicate very likely (90th percentile) changes.

In contrast, human-induced climate change suppressed the occurrence of floods in Asia (Songhua River in 1975 and Mekong River in 1996), South America (Rio Paraguay River in 1980), North America (Mississippi River in 1975), and Europe (Rhine River in 1983 and Elbe River in 1995). The attenuation of these events may be explained by the decreased maximum precipitation in the HPB experiment compared with the NAT experiment (Table 1), as well as increased temperatures in the HPB experiment that reduced the likelihood of snowfall^17^ and decreased the snow melting peak in some snow-affected rivers (e.g., the Mississippi, Rhine, and Elbe Rivers). The reason for the significant suppression in flooding over the two Asian rivers is unclear, as both rivers have similar annual and seasonal (May to August) mean precipitation (Supplementary Fig. 5, Supplementary Fig. 6). Higher evapotranspiration or less snowmelt (Songhua and the upper Mekong) in HPB than NAT could explain this flood suppression.

**Figure 2:**
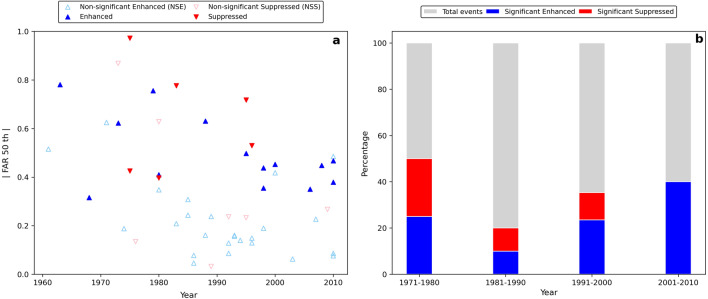
(**a**) Scatterplots of fraction of attribution risk (FAR, absolute values) and flood year and (**b**) bar chart of enhancement or suppression of flooding events during 1961–2010. Detailed information on these flood events is provided in Supplementary Table S1.

More than 50% of flood events shown in Fig. 2 occurred in the most recent 20 years of the study period, partly because more events have been registered in the disaster database in recent years and partly because human-induced climate change altered the probabilities of 20 of 52 analyzed flood events, and that half of these events occurred after 1990. Even ignoring sample size variation among decades, the probability of finding a significant enhancement has been higher in more recent years (Fig. 2b). This illustrates that the increase in flooding has become more prominent in recent years.

Note that this study examined more flood events from the same period (2010**–**2013) than our previous study using MIROC5 AGCM^13^. The difference in the spatial resolutions of the previous study (1.4 degrees) and this study (0.5625 degrees), as well as in the models’ abilities to replicate past climates, may explain why the present study was able to identify a larger number of flood events, as we selected flood events only when the frequency in HBP of flooding (e.g., larger in magnitude than the 1/10 return period) in the specific flood year was larger than that of the average of whole period. Comparing the previous study^13^ and the present study for the same period, both studies indicated that human-induced climate change increased the probability of the Indus River flood in 2010, although this increase was non-significant. By contrast, non-significant enhanced flooding occurred in the Fitzroy River in 2010^13^. These two findings may have resulted from differences in skill and predictability, or the use of different AGCMs to generate large ensemble climate experiments, each of which has developed with its own algorithm to simulate physical atmosphere, ocean, cryosphere, and land surface processes. Conversely, they may have been cause by the application of different methods to generate NAT simulations for these datasets. In a previous study^13^, the 2010 Fitzroy River flood was attributed to climate warming based on 50th percentiles of FAR scores between two different NAT experiments (ALL and NAT_dtr); this result showed the opposite direction of change between the 50th and 90th percentiles, which signaled that the effect of anthropogenic climate change was not strong in this flood event.

The large ensemble discharge simulation used in this study was derived from a single general circulation model, MRI-AGCM3.2, integrated with the CaMa-Flood model. Therefore, the results of this study depend on uncertainties in the hydrologic processes of these models. For example, one study indicated that intense tropical cyclones are not well represented due to the lack of horizontal resolution^14^. The consideration of neglected human water management and biases in AGCMs, and the implementation of a single method for removing past climate change signals in NAT, simulation could add further uncertainty, whereas the simulated discharge in HPB was comparable to the range of variability in the observed discharge (Supplementary Fig. 3, Supplementary Fig. 4).

In summary, it is clear that human-induced climate change has increased the occurrence of extreme river flooding events for the past several decades, mostly in South Asia and South America. Furthermore, a decline in the occurrence of river floods was also identified in some regions (e.g., North America and Europe), possibly due to decreased snowfall with warmer surface temperature. Interestingly, human-induced climate change had a stronger effect on floods that occurred in recent years. This study attempted to quantify the contribution of human-induced climate change on a per-event basis. It provides important insights into adaptation strategies for river flooding for which long-term observations are lacking.

## Methods

### River discharge simulation

To obtain the large ensemble of river discharge, total runoff from the d4PDF experiment data (historical past simulation [HPB] and hypothetical counterfactual natural simulation [NAT]) were input into the river and inundation model CaMa-Flood^15,19^. HPB and NAT each contain 100 ensemble members from 1951 to 2010. HPB was generated with the forcing of historical anthropogenic factors and lower boundary conditions, including the observed monthly mean sea surface temperature (SST), sea ice cover (SIC), and sea ice thickness (SIT). The NAT experiment was generated with the same initial and boundary perturbations as the HPB, but with external forcing factors fixed under pre-industrial conditions, as well as with the exclusion of the warming trend component detected from the SST, SIT, and SIC (see Supplementary Information for the detail of d4PDF experiments)^14^.

The simulated daily river discharges from the CaMa-Flood model have 0.25° × 0.25° resolution. Subsequently, the annual maximum daily discharge was obtained for each ensemble experiment. A time series of annual maximum daily discharge was used for an estimated 10-year return period flood by fitting the Gumbel distribution using the L-moment method^20,21^. The parameters obtained from the HPB were used to calculate return periods at the location and year of the annual maximum daily discharge for the NAT.

The comparison of the annual maximum daily discharge (AMDD) and cumulative distribution function (CDF) of AMDD from 1951–2010 for the observations (GRDC and S14FD discharge reanalysis) and the HPB were conducted to evaluate the representability of the d4PDF-derived discharge data (Fig. S1-S2). Furthermore, we found that historical precipitation events in the HPB experiment had similar spatial patterns to those in previous observational studies, such as over the Yangtze River in 2002^22^, the Indus River basin in 2010^23^, the Amazon River basin in 2009^24^, and the Magdalena River basin in 2008^25^. This indicates that the d4PDF adequately reproduces the climatic field during flooding.

### Selection of flood events

The 52 river flood events that occurred from 1951 to 2010 were identified based on the EM-DAT, news media, and S14FD discharge reanalysis if there were not enough long-term observation records (e.g., GRDC). The detailed procedure for flood event extraction is as follows. First, the top 542 worst cases with respect to the affected population and the top 466 worst cases with respect to economic damage were filtered out, and then 176 cases included under both selection criteria were identified. Second, the flooded river basins were located using additional information (e.g., references and news media), to identify the nearest GRDC observation site. Ultimately, 52 flood events were selected, in which the river basin area upstream of the flooding location was larger than 40,000 km^2^, S14FD discharge reanalysis was strongly correlated with most of the peak discharge derived from the corresponding GRDC^13,26^, and the flood year exceeded the magnitude of a 10-year flood in all available GRDC and S14FD discharge reanalyses and HPB simulations (Supplementary Table 1). Throughout this process, we did not include some historical major flood events that we determined could not be reproduced by our modeling framework (e.g., upstream river size) or locations where historical simulations could not be validated using in situ flow observations.

### Attribution of flood events—fraction of attributable risk (FAR)

The FAR was calculated as *FAR* = *(P*_*HPB−*_*P*_*NAT*_*)/P*_*HPB*,_ where *P*_*HPB*_ and *P*_*NAT*_ are the probability of occurrence of the flood event with anthropogenic changes and without anthropogenic changes, respectively. Uncertainties in FAR values were estimated using the bootstrapping method^27^ (Supplementary Fig. S3). Then, the 10th, 50th, and 90th percentiles of the FAR values from bootstrapped FAR distributions were provided and used to evaluate the attribution of climate change. For example, anthropogenic climate change enhanced (suppressed) river flooding when the 50th and 90th percentiles of the FAR shared the same direction and were opposite in direction from the 10th percentile of FAR. Similarly, anthropogenic climate change enhanced (suppressed) river flooding in a *very likely* range when the 10th, 50th, and 90th percentiles of FAR had the same direction (Table S1). The distributions of these FARs are shown as histograms in Supplementary Figure S4.

## Supplementary Information

Supplementary Information.

## Data Availability

Data generated and analyzed during this study are available for research purposes. Additional datasets used for modeling are available from the corresponding author upon request.
