# Supplementary material for: Enhancement of river flooding due to global warming
Source: Sci Rep. 2022 Nov 30;12:20687. doi: 10.1038/s41598-022-25182-6 (PMC9712344; doi:10.1038/s41598-022-25182-6)
Supplement: Supplementary file 1 — Supplementary Information. [file 41598_2022_25182_MOESM1_ESM.pdf]

Supplementary materials

Enhancement of river flooding due to global warming

Haireti Alifu[1,2*], Yukiko Hirabayashi[1], Yukiko Imada[3], Hideo Shiogama[4]

[1] Department of Civil Engineering, Shibaura Institute of Technology, Tokyo, Japan

[2] Taylor Geospatial Institute, Saint Louis University, United State Email:alifu.haireti@slu.edu

[3] Meteorological Research Institute, Tsukuba, Japan

[4] National Institute for Environmental Studies, Tsukuba, Japan

Summary

S1. Supplementary Data

S2. Supplementary Methods

a. Generation of large ensemble river discharge simulations

b. Evaluation of simulation discharge

c. Attribution of flood events — Fraction of attributable Risk (FAR)

S3. Additional Results

a. Africa

b. Asia

c. Europe

d. North America

e. Oceania

f. South America

S4. References

S1. Supplementary Data

To investigate the contribution of anthropogenic climate change, we used data from the Emergency Events Database (EM-DAT) [1], river discharge data from Global Runoff Data Centre (GRDC), global daily discharge reanalysis (referred to as S14FD discharge reanalysis; derived from the CaMa-Flood [Catchment-based Macro-scale Floodplain] model[2] coupled with a land surface model, MATSIRO[3], under forcing by the S14FD retrospective meteorological forcing dataset[4]), and large ensemble climate experiments based on d4PDF (Database for Policy Decision-Making for Future Climate change)[5] derived from MRI-AGCM3.2.

Predicting climate change and quantitatively assessing the factors associated with its uncertainty are essential for determining the effects of global warming and developing adaptation strategies. However, because abnormal and extreme events have both a low occurrence frequency and large internal variability, it was difficult to adequately evaluate the natural fluctuations based on the currently available prediction databases, since most have relatively small ensemble sizes (<10). Therefore, to better reproduce and evaluate extreme climate events, the d4PDF was released by the Ministry of Education, Climate Change Risk Information Creation Program, and the Japan Agency for Marine-Earth Science and Technology. The large ensemble d4PDF simulations were generated to

investigate the detailed probabilistic distribution of historical climate variability. The d4PDF was generated based on high-resolution AGCMs and was used to conduct a large number of ensemble experiments (up to 100 members), making it possible to reproduce extreme weather conditions at the tail of the probability density distribution. The d4PDF data are available at the Data Integration and Analysis System (DIAS) (http://d4PDF.diasjp.net/).

The simulation database d4PDF has a horizontal resolution of about 60 km[5]. Two large ensembles of human-induced forcing and without-human-induced (natural) forcing experiments were produced in an AGCM-based probabilistic event attribution approach. This study used total runoff from these two types of 100 ensemble climate experiments, which are described below.

Historical past simulation (HPB): Contains 100 ensemble members for the 60 years from 1951 to 2010. HPB was generated with the forcing of historical anthropogenic factors such as the global mean concentration of greenhouse gases and three-dimensional distributions of ozone and aerosol[5]. Moreover, the observed monthly mean sea surface temperature (SST), sea ice cover (SIC) (Centennial Observation-Based Estimates of SST, version 2 [COBE-SST2][6]), and sea ice thickness (SIT) were used as the lower boundary condition[5].

Nonwarming simulation (NAT): The NAT contained the same number of ensembles and periods as the HPB experiment. The NAT experiment was generated with the same initial and boundary perturbations as the HPB, but external forcing factors such as greenhouse gas concentrations, sulfate, black carbon, and organic carbon emissions were fixed under pre-industrial conditions. Distribution of ozone was fixed at the 1960–1962 average, and the warming trend component detected from the SST was excluded (mean of 1900–1919)[5].

S2. Supplementary Methods

a. Generation of large ensemble river discharge simulation

In order to obtain the large ensemble simulation of river discharge, total runoff from the d4PDF experiment data (HPB and NAT) was input into the CaMa-Flood model and parameterized by sub-grid-scale topography based on 1 km-resolution CaMa-Flood topographic datasets[7]. The final results of simulated daily river discharges from the CaMa-Flood model have a $0.25° \times 0.25°$ resolution. The simulation domain was set on a global scale. In this study, we used the default setup of CaMa-Flood version 3.9.6, and the simulation period was the same as d4PDF data with the spin-up procedure of repeating the first year. Subsequently, the annual maximum daily discharge was obtained for each experiment. The time series of annual maximum daily discharge was used for an

estimated 10-year return period flood by fitting the Gumbel distribution using the L-moment method[8,9]. The parameters obtained from the HPB were used to calculate return periods at the same location and year of the annual maximum daily discharge for the NAT.

b. Evaluation of simulation discharge

In total, 52 global flood events were identified (Fig. 1 and Supplementary Table 1). Twenty-seven of the 52 flood events occurred after 1990. The flood events occurred in 32 river basins (Supplementary Table 1). Before assessing the effects of human-induced climate change on these flood events, it is essential to evaluate the representability of the d4PDF derived discharge data. Therefore, a comparison of annual maximum daily discharge (AMDD) and the cumulative distribution function (CDF) of the AMDD during the period of 1951–2010 for the observations (GRDC and S14FD discharge reanalysis[10]) and HPB were conducted (Supplementary Figure 1 and Supplementary Figure 2).

Supplementary Figure 1 illustrates the similarity of peak discharge and potential differences between the d4PDF-derived AMDD and the observations (GRDC and S14FD discharge reanalysis). For the 18 events for which observations were available (red lines in Supplementary Figure 1), S14FD and GRDC had similar magnitudes as AMDD. The S14FD discharge reanalysis exhibited overestimation in the cases of Missouri, Sao Francisco, Rhone, Rhine, Elbe and Sacramento, and underestimation in the Amazon basin.

The overestimation of S14FD discharge reanalysis may be attributable to the fact that the model does not consider human river management activities, irrigation, or land use.

The CDF of the AMDD of flood events indicated that the distribution of the observed (GRDC) AMDD was within the probable range of the HPB experiment (long-term) in 11 basins (Fitzroy, Flinders, Rhine, Elbe, Missouri, Ohio, Mississippi, and Susquehanna River basins) of the 18 basins for which long-term observations were available (black lines in Supplementary Fig. 2). The remaining seven basins (the Niger, Lena, Amur, Chao Phraya, Nam Mum, Amazon, and Sao Francisco River basins) exhibited overestimation in the HPB experiment compared to GRDC. Figure 2 also illustrates that most of the distribution of CDF of the AMDD S14FD discharge reanalysis was within the probable range of the HPB experiment (long-term), except in the Niger, Xijiang, Indus, Karun, Magdalena, Parana, Rio Paraguay, Amazon, Rhone, Rhine, and Elbe River basins. Most of these basins also showed smaller annual fluctuations in the AMDD, perhaps due to human water management. Since we did not remove the biases of climatic variables from the AGCM, the errors also reflect errors in variables such as precipitation and land surface processes, including evapotranspiration and infiltration. Despite the biases in magnitude in these basins, the HPB experiment reasonably reproduced the fluctuations in the annual maximum daily discharge in most basins.

We also found that representability of d4PDF derived discharge from Fitzroy (5101301), Peace (4208450), Amur (2906700), Mississippi (4127501), and Murrumbidgee (5204106) river basins were not as good as a past study[11] which used different large ensemble climate experiment. However, we also found that Indus River historical discharge patterns from the HPB experiment (this study) had a more similar spatial pattern with reanalyzed discharge compared to the previous study[11]**.** CDF of annual maximum daily discharge also indicates that results from this study are at least somehow within the 5-95 percentile range of HPB simulation compared to the past study.

c. Attribution of flood events — Fraction of Attributable Risk (FAR)

The fraction of attributable risk (FAR) method was used to assess the difference in the probability of flood extremes between the HPB and NAT. FAR was proposed by Allen 2003[12], and can be interpreted as an estimate of the probability of necessary causation (by anthropogenic forcing) of an event. The FAR is calculated as:

$$FAR = (P_{HPB} - P_{NAT}) / P_{HPB} \qquad (1)$$

Where PHPB and PNAT are the probability of occurrence of a flood event with anthropogenic changes and without anthropogenic changes, respectively

When the FAR is positive (negative), it means that global warming has enhanced (suppressed) the occurrence of the flood. If the FAR is close to zero, the contribution of

global warming to floods is negligible. Uncertainties in FAR values were estimated using the bootstrapping method[13]. The bootstrap resampling model generated FAR values 1,000 times based on randomly selected available model simulations with replication. Then, the FAR values corresponding to the 50th and 90th percentiles of values in the bootstrapped FAR distributions were provided. The effects of anthropogenic climate change on flood events (E, enhanced; S, suppressed;) were judged as E (S) when both (FAR) scores were between the 50th and 90th percentile of FAR and had the same direction. To analyze the uncertainty of the FAR, the lowest 10th percentiles of the FAR values were checked for the case of positive values obtained in FAR 50th and FAR 90th. For example, river floods for which the 50th and 90th percentiles of FAR had the same direction, but the opposite direction of the 10th percentile of FAR, were classified as non-significantly affected by anthropogenic climate change. We found that human-induced climate change enhanced (suppressed) river floods in a ***very likely*** range (with 90% significance) when the 10th, 50th, and 90th percentiles of the FAR have the same direction (Supplementary Table 1).

S3. Additional results

Using the uncertainty range of the FAR generated from bootstrapping results (Supplementary Fig. 3) and the CDF/PDF (cumulative and probability distribution function) of the AMDD (Supplementary Fig. 4), we evaluated the effects of human-

induced climate change on the occurrence of flooding (Supplementary Table 1). Among the 52 selected flood events for the period of 1951–2010, anthropogenic radiative forcing increased the occurrence of 39 flood events (14 events significantly enhanced), most of which occurred in Asia (13 flood events) and South America (12 flood events), followed by five flood events determined in North America and three flood events in Oceania and Europe (Fig. 1, Supplementary Fig. 4 and Supplementary Table 1). Conversely, the occurrence of 13 flood events was suppressed (six events were significantly suppressed). These included four flood events in North America, three flood events in Asia and Europe, two flood events in Oceania, and one flood event in South America.

a. Africa

Three flood events (caused by heavy rainfall) were identified in the Africa region (Fig. 1 and Supplementary Table 1), and human-induced climate change *very likely* enhanced (significantly) the flood in the Niger River (FAR 0.63/0.78/0.88) in 1963. The Limpopo River floods in 1961 (FAR -0.14/0.52/0.85) and 1996 (FAR -0.83/0.15/0.65) were non-significantly enhanced.

b. Asia

In Asia (Fig. 1-2 and Supplementary Table 1), heavy rainfall and the monsoon effect were the main causes of the flood events. El Niño and storms also caused river flooding in the

Yangtze River in 1998, Mekong River in 1996, and Lena River in 1992. In the NAT experiments, the mean CDF/PDFs of the HPB experiment corresponding to the 13 of 16 flood events were all shifted to a warmer climate (Supplementary Fig. 4 and Supplementary Table 1). The FAR values (Supplementary Table 1) indicated that human-induced climate change ***very likely*** enhanced the Yangtze River flood in 1998 (FAR 0.17/0.36/0.51), the Indus River flood in 1988 (FAR 0.45/0.63/0.75), the Brahmaputra River flood in 1998 (FAR 0.16/0.44/0.61), the Tapi River flood in 1968 (FAR 0.08/0.32/0.50), the Chao Phraya River flood in 1995 (FAR 0.29/0.50/0.64), and the Chao Phraya River flood in 2010 (FAR 0.14/0.47/0.67). Non-significant enhancement was found for the Num Mum River flood in 2000 (FAR -0.13/0.42/0.71), the Ganjiang River flood in 2010 (-0.40/0.08/0.44), the Xijiang River flood in 1998 (FAR -0.08/0.19/0.40), the Indus River flood in 2007 (FAR -0.23/0.23/0.54), the Indus River flood in 2010 (FAR -0.49/0.09/0.44), the Karun River flood in 1980 (FAR -0.27/0.35/0.68), the Lena River flood in 1992 (FAR -0.28/0.09/0.37), and the Amur River flood in 1985 (FAR -0.04/0.24/0.47).

The results of FAR indicated that human-induced climate change suppressed the occurrence of flooding in the Songhua River flood in 1975 (FAR -2.35/-0.97/-0.12) and the Mekong River flood in 1996 (FAR -1.24/-0.53/-0.10). Non-significant suppression

was found for the Ganjiang River flood in 1992 (FAR -0.83/-0.24/0.18).

c. Europe

In Europe (Fig. 1), human-induced warming was found to have very likely suppressed the Rhine River flood in 1983 (FAR -1.62/-0.78/-0.19) and the Elbe River flood in 1995 (FAR -1.49/-0.72/-0.20)]. Heavy rainfall was the main cause of floods in this region. The Rhone River flood in 1994 (FAR -0.30/0.14/0.45), the Rhine River flood in 1993 (FAR -0.32/0.16/0.47), and the Elbe River flood in 2003 (FAR -0.27/0.06/0.33) were non-significantly enhanced.

d. North America

In North America (Fig. 1), the results indicated that the Mississippi River flood in 1975 (FAR -0.89/-0.43/-0.10) was ***very likely*** suppressed by warming. Non-significant enhancement was observed in the Missouri River flood in 1986 (FAR -0.35/0.08/0.38), the Missouri River flood in 1993 (FAR -0.23/0.16/0.44), the Ohio River flood in 1993 (FAR -0.15/0.24/0.50), the Mississippi River flood in 1986 (FAR -0.28/0.05/0.31), and the Susquehanna River flood in 1996 (FAR -0.25/0.13/0.21) (Supplementary Fig. 4).

Human-induced climate change ***very likely*** suppressed flooding in the Mississippi River in 1975 (FAR -0.89/-0.43/-0.10). Non-significant suppression was observed for the Sacramento River flood in 1980 (FAR -1.95/-0.63/0.02), the Peace River flood in 1996

(FAR -0.25/0.13/0.39), and the Peace River flood in 2009 (FAR -0.87/-0.27/0.21). Heavy rainfall and additional snowmelt (Susquehanna River flood in 1996 and Peace River flood in 1976) were the main causes of floods in North America.

e. Oceania

There was no significant enhancement or suppression of flood events in Oceania (Fig. 1). The Fitzroy River flood in 2010 (FAR -1.76/0.48/0.94) and the Flinders River floods in 1971 (FAR -0.27/0.62/0.93) and 1974 (FAR -0.99/0.19/0.65) were non-significantly enhanced, and the Fitzroy River flood in 1973 (FAR -5.11/-0.87/0.38) and the Murrumbidgee River flood in 1989 (FAR -2.14/-0.03/0.71) were non-significantly suppressed. Heavy rainfall and La Niña-induced heavy rainfall were the main cause of floods in this region.

f. South America

In South America (Fig. 1 and Supplementary Table 1), human-induced climate change very likely enhanced the Magdalena River flood in 1973 (FAR 0.49/0.62/0.72), the Magdalena River flood in 2008 (FAR 0.17/0.45/0.63), the Magdalena River flood in 2010 (FAR 0.11/0.38/0.58), the Amazon River flood in 2000 (FAR 0.16/0.45/0.65), the Amazon River flood in 2006 (FAR 0.20/0.35/0.47), the Sao Francisco River flood in 1979 (FAR 0.60/0.76/0.85), and the Sao Francisco River flood in 1980 (FAR 0.08/0.41/0.61).

It is clear that the mean CDF/PDFs of the HPB experiment for the Magdalena, Parana, Amazon, and Sao Francisco River flood events shifted to the right (higher in magnitude in HPB than that in NAT) (Supplementary Fig. 4). Non-significant enhancement was observed for the Magdalena River flood in 1988 (FAR -0.03/0.16/0.31), the Parana River flood in 1983 (FAR -0.08/0.21/0.43), the Parana River flood in 1992 (FAR -0.29/0.13/0.39), and the Sao Francisco River flood in 1985 (FAR -0.41/0.31/0.70). Human-induced climate change very likely suppressed the Rio Paraguay River flood in 1980 (FAR -0.90/-0.40/-0.001). Heavy rain was the main cause of flood events in South America. Moreover, La Niña and El Nino events affected the flood extremes in the Magdalena River and Parana River floods.

References

1 EM-DAT. *The OFDA/CRED international disaster database*, <http:// www.emdat.be.> (

2 Yamazaki, D., Kanae, S., Kim, H. & Oki, T. A physically based description of floodplain inundation dynamics in a global river routing model. *Water Resour. Res.* **47**, doi:10.1029/2010wr009726 (2011).

3 Takata, K., Emori, S. & Watanabe, T. Development of the minimal advanced treatments of surface interaction and runoff. *Global Planet. Change* **38**, 209-222, doi:10.1016/S0921-8181(03)00030-4 (2003).

4 Iizumi, T., Takikawa, H., Hirabayashi, Y., Hanasaki, N. & Nishimori, M. Contributions of different bias-correction methods and reference meteorological forcing data sets to uncertainty in projected temperature and precipitation extremes. *Journal of Geophysical Research-Atmospheres* **122**, 7800-7819, doi:10.1002/2017JD026613 (2017).

5 Mizuta, R. *et al.* Over 5,000 years of ensemble future climate simulations by 60-km global and 20-km regional atmospheric models. *BAMS* **98**, 1383-1398 (2017).

6 Hirahara, S., Ishii, M. & Fukuda, Y. Centennial-Scale Sea Surface Temperature Analysis and Its Uncertainty. *Journal of Climate* **27**, 57-75, doi:10.1175/jcli-d-12-00837.1 (2014).

7 Yamazaki, D. *et al.* MERIT Hydro: A high‐resolution global hydrography map based on latest topography dataset. *Water Resour. Res.* **55**, 5053-5073 (2019).

8 Gumbel, E. J. The return period of flood flows. *The annals of mathematical statistics* **12**, 163-190 (1941).

9 Hosking, J. R. L‐moments: Analysis and estimation of distributions using linear combinations of order statistics. *Journal of the Royal Statistical Society: Series B (Methodological)* **52**, 105-124 (1990).

10 Tanoue, M., Taguchi, R., Alifu, H. & Hirabayashi, Y. Residual flood damage under intensive adaptation. *Nature Clim. Change* **11**, 823-826, doi:10.1038/s41558-021-01158-8 (2021).

11 Hirabayashi, Y. *et al.* Anthropogenic climate change has changed frequency of past flood during 2010-2013. *Progress in Earth and Planetary Science* **8**, 1-9 (2021).

12 Allen, M. Liability for climate change. *Nature* **421**, 891-892 (2003).

13 Efron, B. & Tibshirani, R. J. *An introduction to the bootstrap*. (CRC press, 1994).

14 Zwarts, L. I. & Frerotte, J. *Water crisis in the Inner Niger Delta (Mali): causes, consequences, solutions*. (Altenburg & Wymenga ecologisch onderzoek bv, 2012).

15 Maposa, D. & Cochran, J. J. Modelling extreme flood heights in the lower Limpopo River basin of Mozambique using a time-heterogeneous generalised Pareto distribution. *Statistics and Its Interface* **10**, 131-144 (2017).

16 Mathivha, F., Nkosi, M. & Mutoti, M. Evaluating the relationship between hydrological extremes and groundwater in Luvuvhu River Catchment, South Africa. *Journal of Hydrology: Regional Studies* **37**, 100897 (2021).

17 Li, H., Bao, S., Wang, X. & Lv, H. Storm flood characteristics and identification of periodicity for flood-causing rainstorms in the second Songhua River Basin. *Water* **8**, 529 (2016).

18 Shankman, D., Keim, B. D. & Song, J. Flood frequency in China's Poyang Lake region: trends and teleconnections. *International Journal of Climatology: A Journal of the Royal Meteorological Society* **26**, 1255-1266 (2006).

19 Brown, A. Invisible People, Pollution, and Places: Nuclear Contamination on the Tibetan Plateau, Himalayan Rivers, and Water Users. *East Asia's Renewed Respect for the Rule of Law in the 21st Century: The Future of Legal and Judicial Landscapes in East Asia* **12**, 282 (2015).

20 Wu, Y.-J., Gough, W., Jiang, T. & Kung, H.-T. The variation of floods in the middle reaches of the Yangtze River and its teleconnection with El Niño events. *Advances in Geosciences* **6**, 201-205 (2006).

21 Brakenridge, G. (2018).

22 Carling, P. *et al.* Are equilibrium multichannel networks predictable? The case of the regulated Indus River, Pakistan. *Geomorphology* **302**, 20-34 (2018).

23 Ali, A. Indus basin floods: Mechanisms, impacts, and management. (2013).

24 Syvitski, J. P. & Brakenridge, G. R. Causation and avoidance of catastrophic flooding along the Indus River, Pakistan. *GsA today* **23**, 4-10 (2013).

25 Dezfuli, A. Rare atmospheric river caused record floods across the Middle East. *BAMS* **101**, E394-E400 (2020).

26 Rumbia, T. S. & Joe, T. Annual Mekong Flood Report 2013.

27 Dhar, O. & Nandargi, S. A study of floods in the Brahmaputra basin in India. *International Journal of Climatology: A Journal of the Royal Meteorological Society* **20**, 771-781 (2000).

28 Ramaswamy, C. & RAO, V. S. Severe and catastrophic floods in the Tapi and the Narmada. *Curr. Sci.*, 375-379 (1980).

29 Gautier, E. *et al.* Going with the flow: Hydrologic response of middle Lena River (Siberia) to the climate variability and change. *J. Hydrol.* **557**, 475-488 (2018).

30 Kim, V. in *Report on Amur-Okhotsk Project* 129-138 (2010).

31 Hungspreug, S., Khao-uppatum, W. & Thanopanuwat, S. Flood management in Chao Phraya river basin. *The Chao Phraya Delta* **293** (2000).

32 Sawano, H., Kuribayashi, D. & Hagiwara, Y. Lessons Learned from the Flood Disaster in Industrial Estates/Parks/Zones in Thailand. *Technical Note of PWRI* (2016).

33 Goteti, G. & Lettenmaier, D. P. *Effects of streamflow regulation and land cover change on the hydrology of the Mekong river basin*, University of Washington, (2001).

34 Goez, H. H. Flood management and slums formation in Magdalena? s River Basin-Colombia. *Retrieved from* (2005).

35 Enciso, A. M., Carvajal-Escobar, Y. & Sandoval, M. C. Hydrological analysis of historical floods in the upper valley of Cauca river: Análisis hidrológico de las crecientes históricas del río Cauca en su valle alto. *Ingeniería y competitividad* **18**, 47-58 (2016).

36 Camilloni, I. & Barros, V. The Parana river response to El Nino 1982–83 and 1997–98 events. *Journal of Hydrometeorology* **1**, 412-430 (2000).

37 de Resende, A. F. *et al.* Massive tree mortality from flood pulse disturbances in Amazonian floodplain forests: The collateral effects of hydropower production. *Sci Total Environ* **659**, 587-598 (2019).

38 Holanda, F. S. R. *et al.* Environmental perception of the São Francisco riverine population in regards to flood impact. *Journal of Human Ecology* **28**, 37-46 (2009).

39 Affairs, U. N. O. f. t. C. o. H. Brazil – Floods Jan 1985 UNDRO Information Report No.1.

40 KUHLMANN, L. G. *An integrated flood damage assessment in Brazil*, (2018).

41 Barros, V., Chamorro, L., Coronel, G. & Baez, J. The major discharge events in the Paraguay River: Magnitudes, source regions, and climate forcings. *Journal of Hydrometeorology* **5**, 1161-1170 (2004).

42 Service, N. W. Historic flood events in the Missouri river basin. (NOAA's National Weather Service).

43 Larson, L. W. The great USA flood of 1993. *IAHS Publications-Series of Proceedings and Reports-Intern Assoc Hydrological Sciences* **239**, 13-20 (1997).

44 McClain, D. L. Flood of February 1989 in Kentucky. Report No. 2331-1258, (Dept. of the Interior, US Geological Survey; Books and Open-File Reports …, 1990).

45 Camillo, C. A. *Divine providence: The 2011 flood in the Mississippi River and tributaries project*. (Mississippi River Commission, 2012).

46 Thompson, J. Early reclamation and abandonment of the central Sacramento-San Joaquin Delta. *Sacramento History Journal* **6**, 41-72 (2006).

47 Service, N. W. The Winter Flood of January 1996.

48 Smith, D. G. in *The 12th workshop on the hydraulics of ice covered rivers. Canadian Geophysical Union-Hydrology Section, Edmonton.* 241-260.

49 Canada, P. S. (2007).

50 Holmes, J. C., QC, M. J. O. S. & Cummins, M. P. RE: Queensland Floods Commission of Inquiry. (2011).

51 Commonwealth of Australia , B. o. M. ***Queensland flood summary 1970-1979***.

52 MDBA. (Murray–Darling Basin Authority Canberra, 2010).

53 ESA), E. S. A.

54 Vervuren, P., Blom, C. & De Kroon, H. Extreme flooding events on the Rhine and the survival and distribution of riparian plant species. *Journal of ecology* **91**, 135-146 (2003).

55 Linnerooth-Bayer, J., Quijano-Evans, S., Lofstedt, R. & Elahi, S. Tsunami Project on the Uninsured Elements of Natural Catastrophic Losses. *International Institute of Applied Systems Analysis, Laxenburg, Austria* (2000).

56 Surminski, S., Roezer, V. & Golnaraghi, M. *Flood Risk Management in Germany: Building Flood Resilience in a Changing Climate*. (Geneva Association-International Association for the Study of Insurance …, 2020).

57 Gierk, M. & de Roo, A. The impact of retention polders, dyke-shifts and reservoirs on discharge in the Elbe river. *JRC Scientific and Technical Report JRC* **49172** (2008).

Supplementary Table 1. Summary of selected flood events with significant effects of human-induced climate change (E, enhanced; S, suppressed). The effects of human-induced climate change on flood events were judged as E (S) when all fraction of attribution (FAR) scores are plus (minus).

	Region	River	GRDC	Year	Causes	FAR			Effect
						FAR^{10th}	FAR^{50th}	FAR^{90th}	
1	AF	NIGER	1134100	1963	HR[14]	0.63	0.78	0.88	E*
2	AF	LIMPOPO	1896501	1961	HR[15]	-0.14	0.52	0.85	E
3	AF	LIMPOPO	1896501	1996	HR[16]	-0.83	0.15	0.65	E
4	AS	SONGHUA	2106600	1975	HR[17]	-2.35	-0.97	-0.12	S*
5	AS	GANJIANG	2181850	1992	HR[18]	-0.83	-0.24	0.18	S
6	AS	GANJIANG	2181850	2010	HR[19]	-0.40	0.08	0.44	E
7	AS	YANGTZE	2181900	1998	HRE[20]	0.17	0.36	0.51	E*
8	AS	XIJIANG	2186800	1998	HR[21]	-0.08	0.19	0.40	E
9	AS	INDUS	2335200	2007	HRM[22]	-0.23	0.23	0.54	E
10	AS	INDUS	2335200	2010	HRM[23]	-0.49	0.09	0.44	E
11	AS	INDUS	2335950	1988	HR[24]	0.45	0.63	0.75	E*
12	AS	KARUN	2423500	1980	HR[25]	-0.27	0.35	0.68	E
13	AS	MEKONG	2569005	1996	HRS[26]	-1.24	-0.53	-0.10	S*
14	AS	BRAHMAPUTRA	2851300	1998	HRM[27]	0.16	0.44	0.61	E*
15	AS	TAPI	2853300	1968	HR[28]	0.08	0.32	0.50	E*
16	AS	LENA	2903427	1992	HRS[29]	-0.28	0.09	0.37	E
17	AS	AMUR	2906700	1985	HR[30]	-0.04	0.24	0.47	E
18	AS	CHAO PHRAYA	2964130	1995	HR[31]	0.29	0.50	0.64	E*
19	AS	CHAO PHRAYA	2964130	2010	HR[32]	0.14	0.47	0.67	E*
20	AS	NAM MUN	2969200	2000	HR[33]	-0.13	0.42	0.71	E
21	SA	MAGDALENA	3103300	1973	HR[34]	0.49	0.62	0.72	E*
22	SA	MAGDALENA	3103300	1988	HRL[35]	-0.03	0.16	0.31	E
23	SA	MAGDALENA	3103300	2008	HRL[35]	0.17	0.45	0.63	E*
24	SA	MAGDALENA	3103300	2010	HRL[35]	0.11	0.38	0.58	E*
25	SA	PARANA	3265601	1983	HRE[36]	-0.08	0.21	0.43	E
26	SA	PARANA	3265601	1992	HRE[36]	-0.29	0.13	0.39	E
27	SA	AMAZON	3618000	2000	HR[37]	0.16	0.45	0.65	E*
28	SA	AMAZON	3618000	2006	HR[37]	0.20	0.35	0.47	E*
29	SA	SAO FRANCISCO	3651800	1979	HR[38]	0.60	0.76	0.85	E*
30	SA	SAO FRANCISCO	3651800	1985	HR[39]	-0.41	0.31	0.70	E
31	SA	SAO FRANCISCO	3651805	1980	HR[40]	0.08	0.41	0.61	E*
32	SA	RIO PARAGUAY	3667060	1980	HR[41]	-0.90	-0.40	-0.001	S*
33	NA	MISSOURI	4122900	1986	HR[42]	-0.35	0.08	0.38	E
34	NA	MISSOURI	4122902	1993	HR[43]	-0.23	0.16	0.44	E
35	NA	OHIO	4123050	1989	HR[44]	-0.15	0.24	0.50	E
36	NA	MISSISSIPPI	4127501	1986	HR[45]	-0.28	0.05	0.31	E
37	NA	MISSISSIPPI	4127800	1975	HR[45]	-0.89	-0.43	-0.10	S*
38	NA	SACRAMENTO	4146280	1980	HR[46]	-1.95	-0.63	0.02	S
39	NA	SUSQUEHANNA	4147703	1996	HRSM[47]	-0.25	0.13	0.39	E
40	NA	PEACE	4208450	1976	HRSM[48]	-0.62	-0.13	0.21	S

41	NA	PEACE	4208450	2009	HR[49]	-0.87	-0.27	0.14	S
42	OC	FITZROY	5101301	1973	HRL[50]	-5.11	-0.87	0.38	S
43	OC	FITZROY	5101301	2010	HRL[50]	-1.76	0.48	0.94	E
44	OC	FLINDERS	5109151	1971	HR[51]	-0.27	0.62	0.93	E
45	OC	FLINDERS	5109151	1974	HR[51]	-0.99	0.19	0.65	E
46	OC	MURRUMBIDGEE	5204106	1989	HR[52]	-2.14	-0.03	0.71	S
47	EU	RHONE	6139100	1994	HRS[53]	-0.30	0.14	0.45	E
48	EU	RHINE	6335200	1983	HR[54]	-1.62	-0.78	-0.19	S*
49	EU	RHINE	6335200	1993	HR[55]	-0.32	0.16	0.47	E
50	EU	ELBE	6340120	1995	HR[56]	-1.46	-0.72	-0.20	S*
51	EU	ELBE	6340150	1995	HR[56]	-0.74	-0.23	0.13	S
52	EU	ELBE	6340150	2003	HR[57]	-0.27	0.06	0.33	E

Note: AF, Africa; AS, Asia; EU, Europe; SA, South America; NA, North America; OC, Oceania; N/A, not available. HR, Heavy rain; M, monsoon; E, El Nino; S, Strom; L, La Niña; SM, Snowmelt; asterisks indicate ***very likely* (90th)** changes***;*** Numbers in Causes columns are representing the reference number.

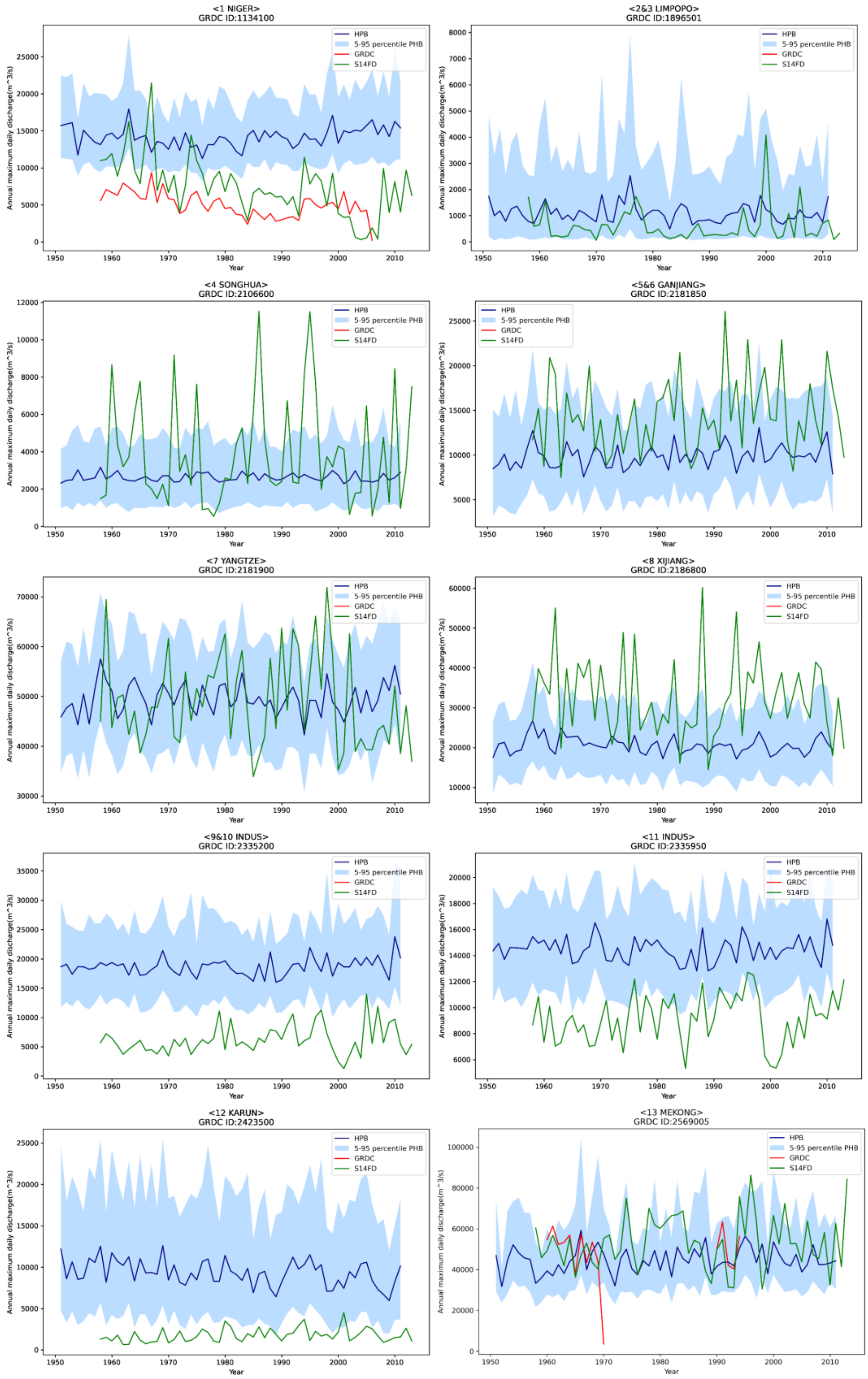

Figure S1. Comparison of annual maximum daily discharge from 1951–2010 for the GRDC, S14FD discharge reanalysis, and HPB (Continuing…).

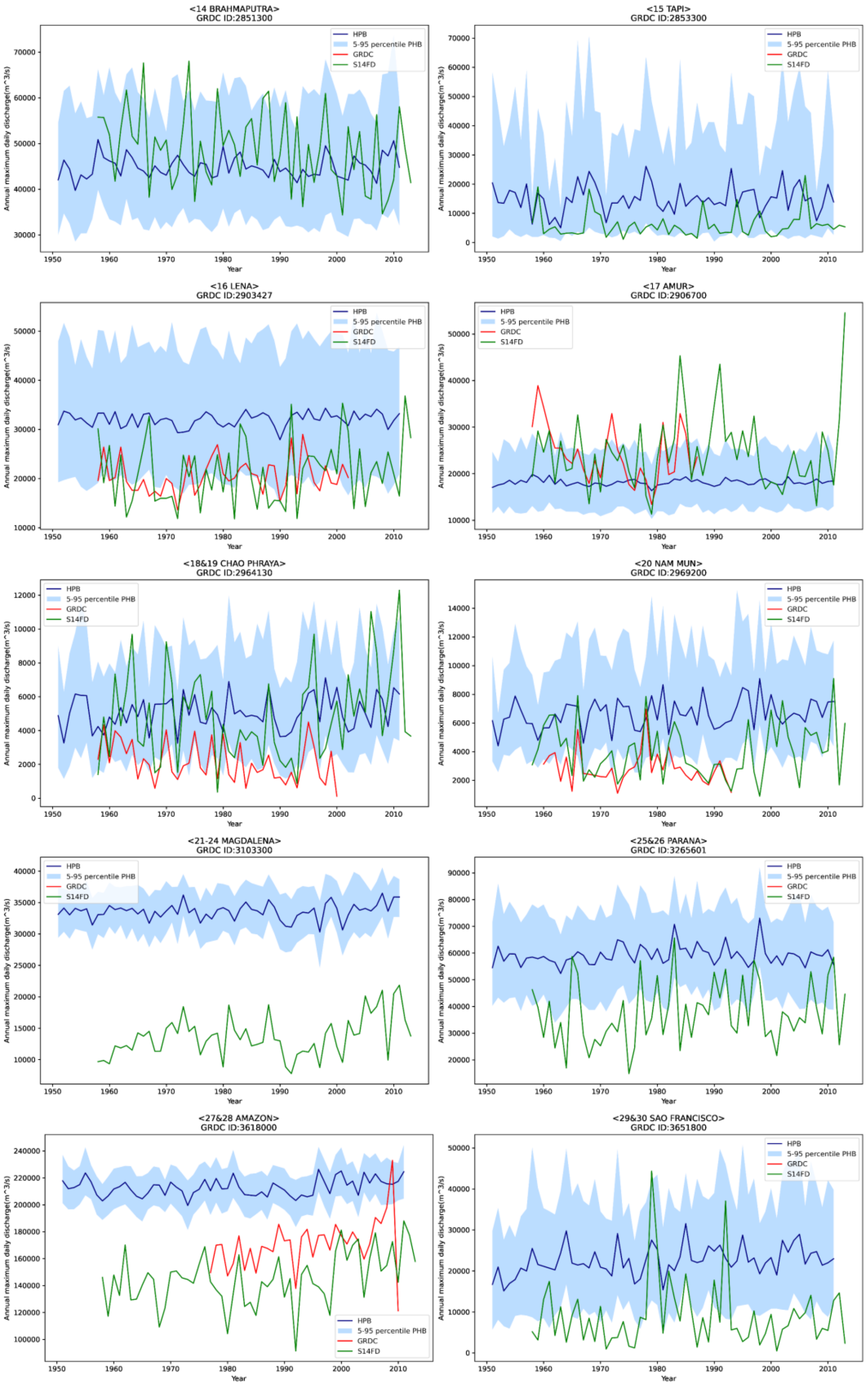

Figure S1. Comparison of annual maximum daily discharge from 1951–2010 for the GRDC, S14FD discharge reanalysis, and HPB (Continuing…).

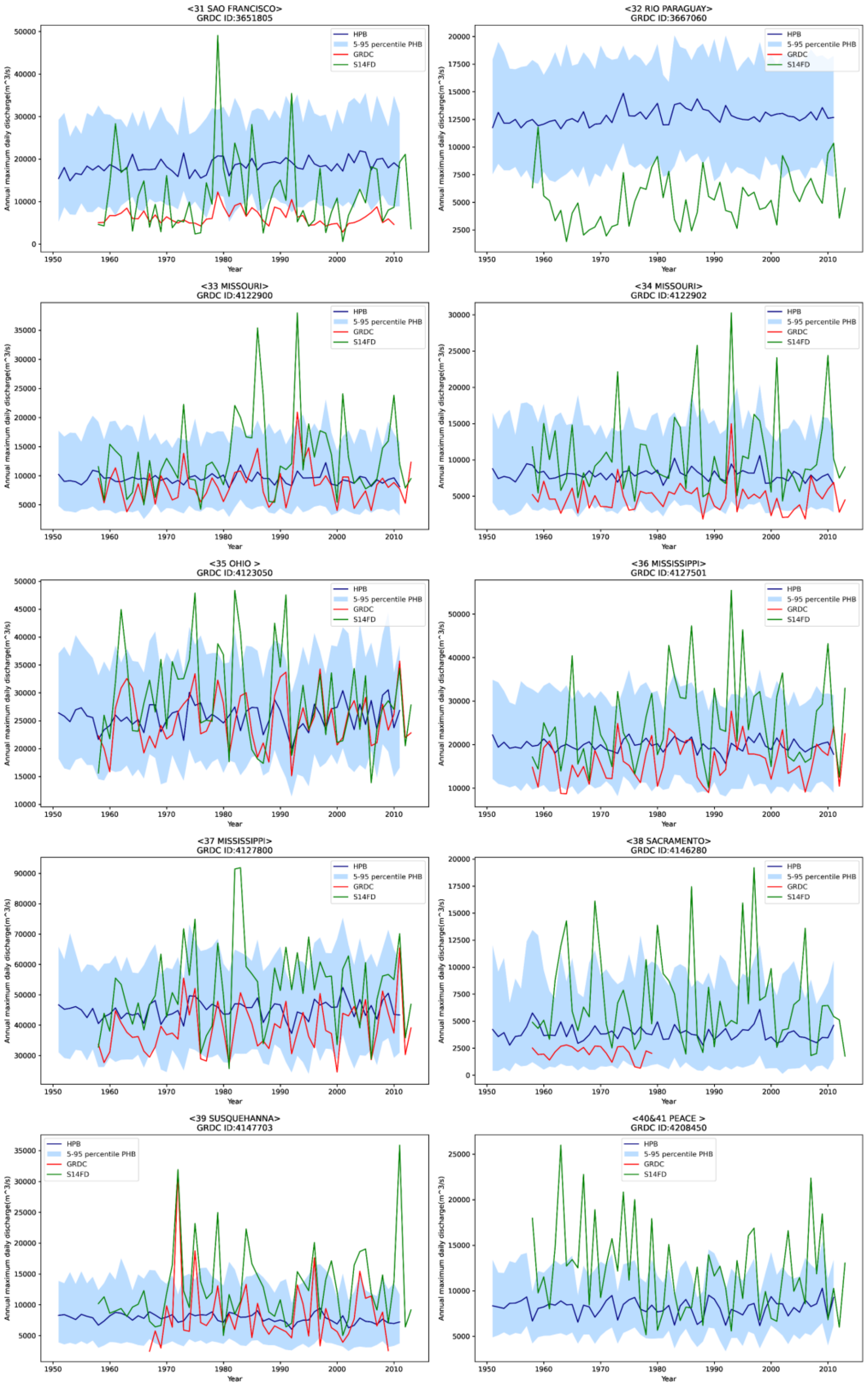

Figure S1. Comparison of annual maximum daily discharge from 1951–2010 for the GRDC, S14FD discharge reanalysis, and HPB (Continuing…).

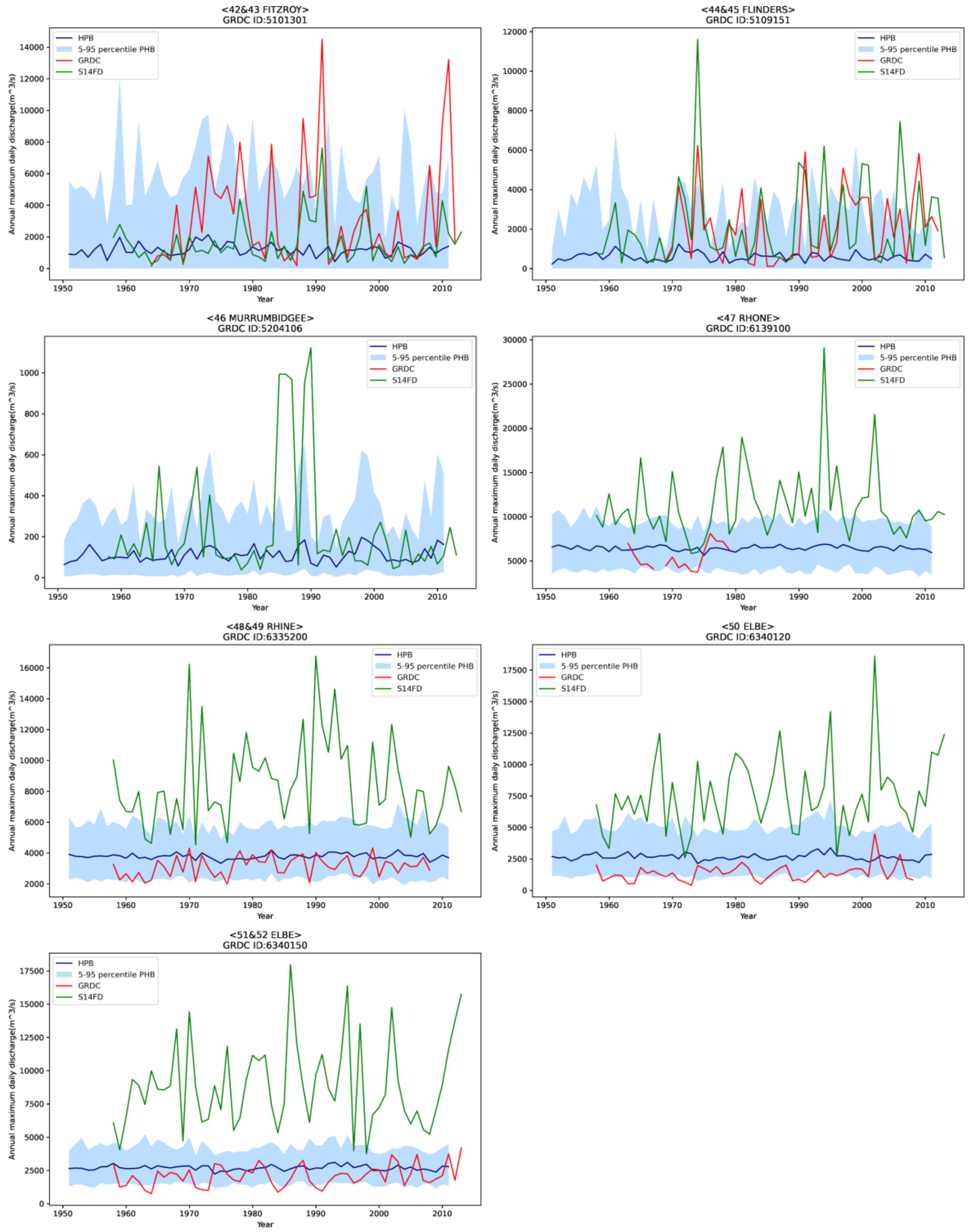

Figure S1. Comparison of annual maximum daily discharge from 1951–2010 for the GRDC, S14FD discharge reanalysis, and HPB.

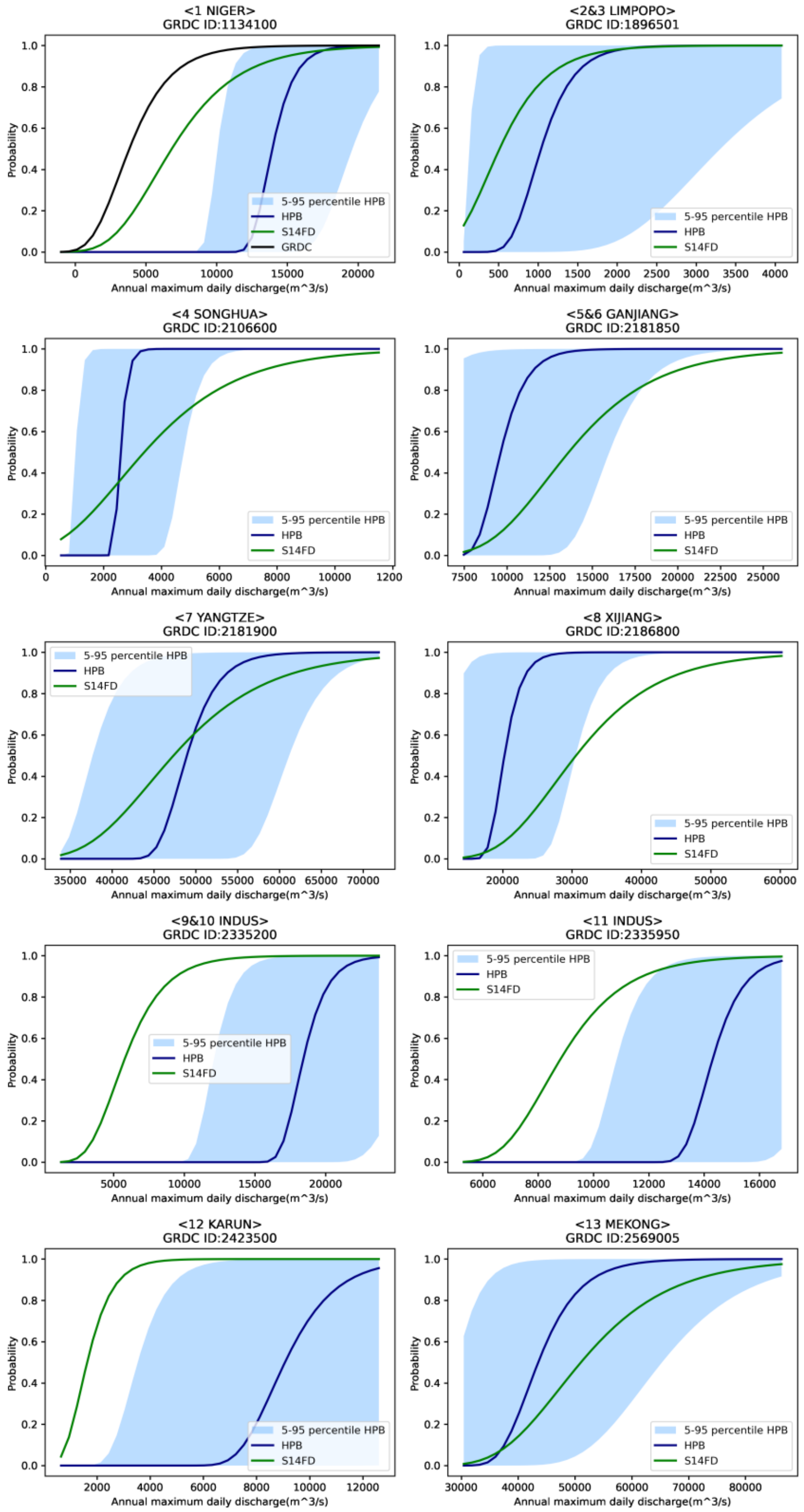

Figure S2. Comparison of cumulative density function (CDF) of annual maximum daily discharge from 1951–2010 for the GRDC, S14FD discharge reanalysis, and HPB (continuing...).

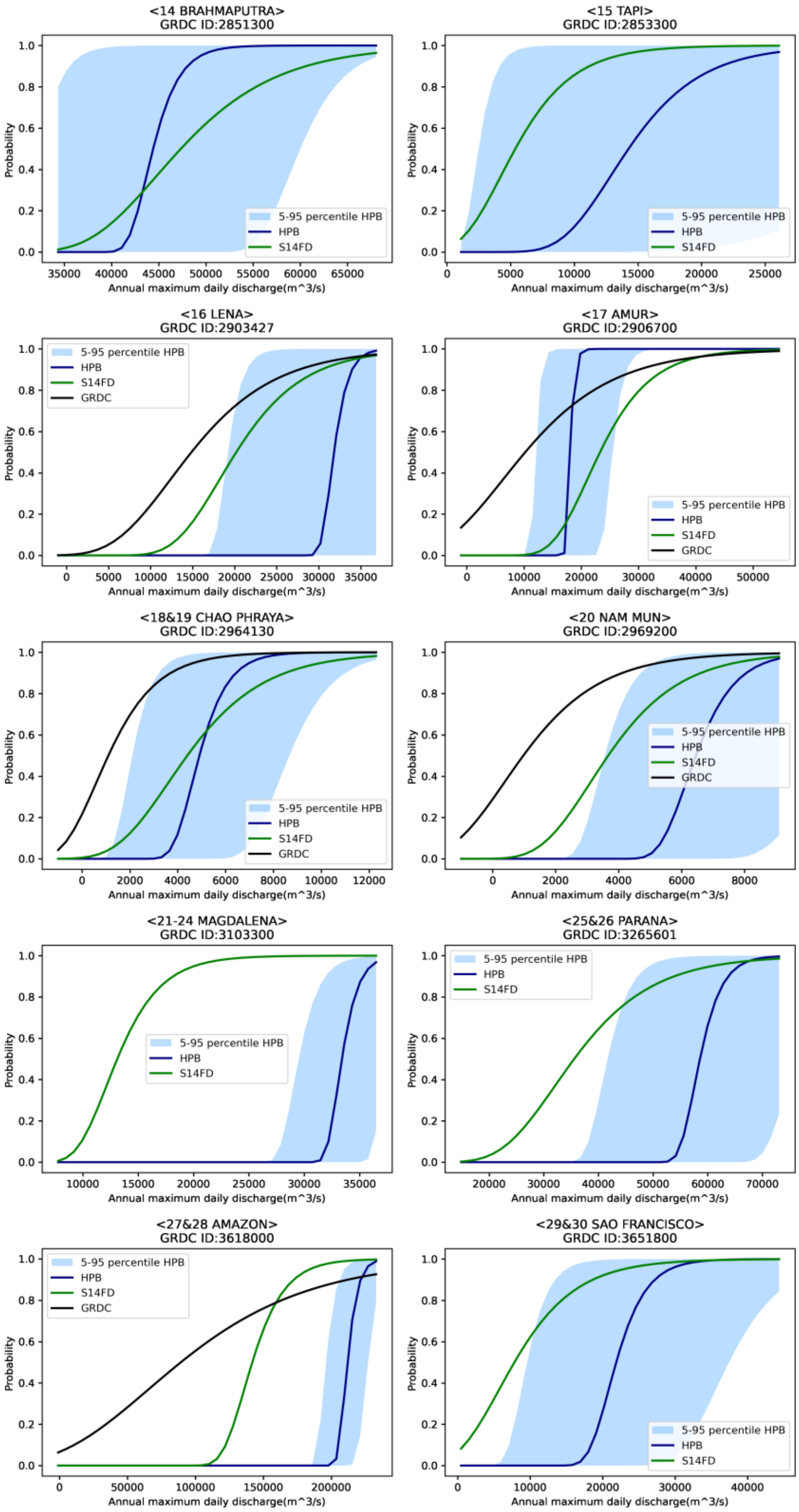

Figure S2. Comparison of cumulative density function (CDF) of annual maximum daily discharge from 1951–2010 for the GRDC, S14FD discharge reanalysis, and HPB (continuing...).

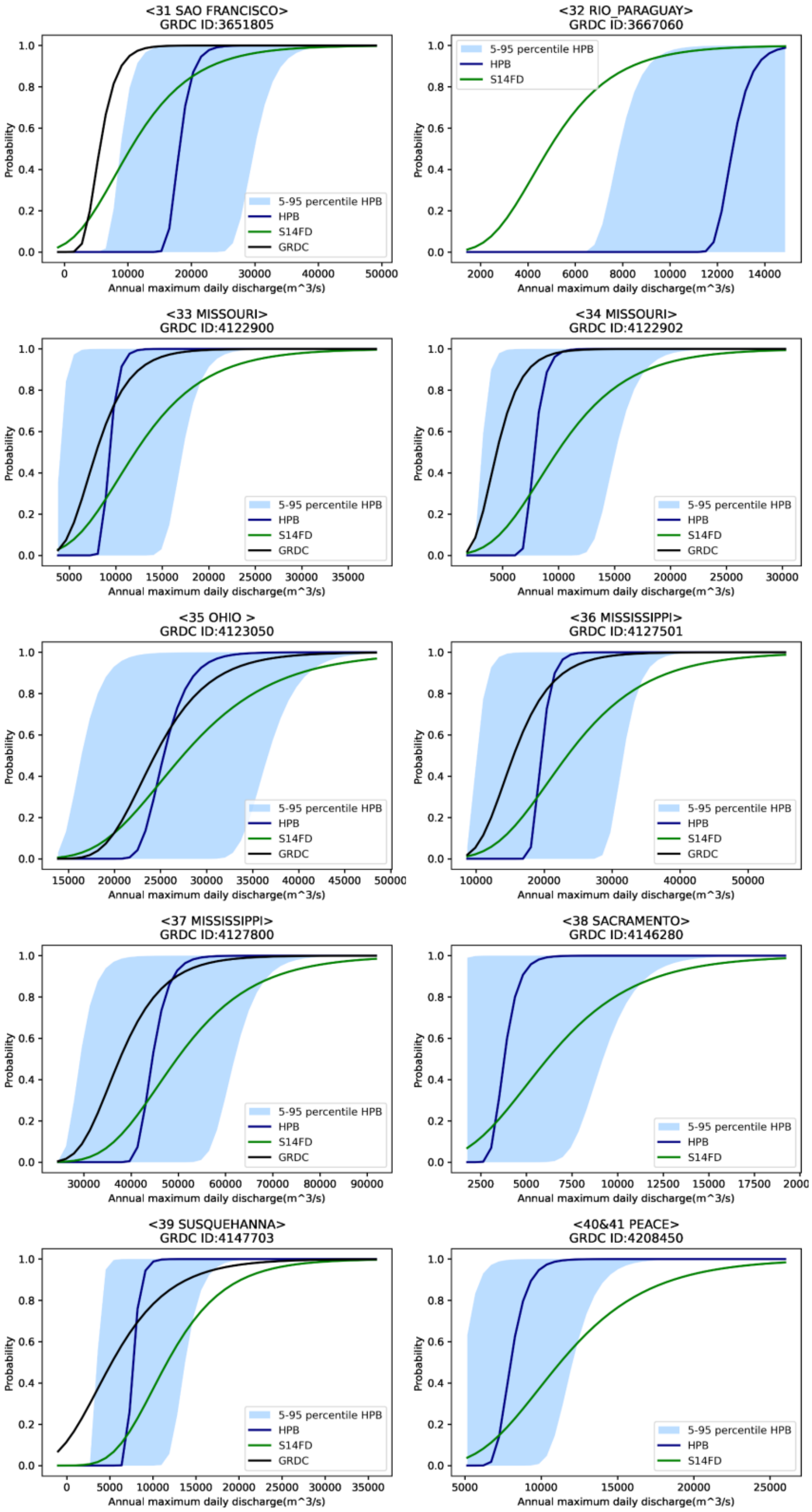

Figure S2. Comparison of cumulative density function (CDF) of annual maximum daily discharge from 1951–2010 for the GRDC, S14FD discharge reanalysis, and HPB (continuing...).

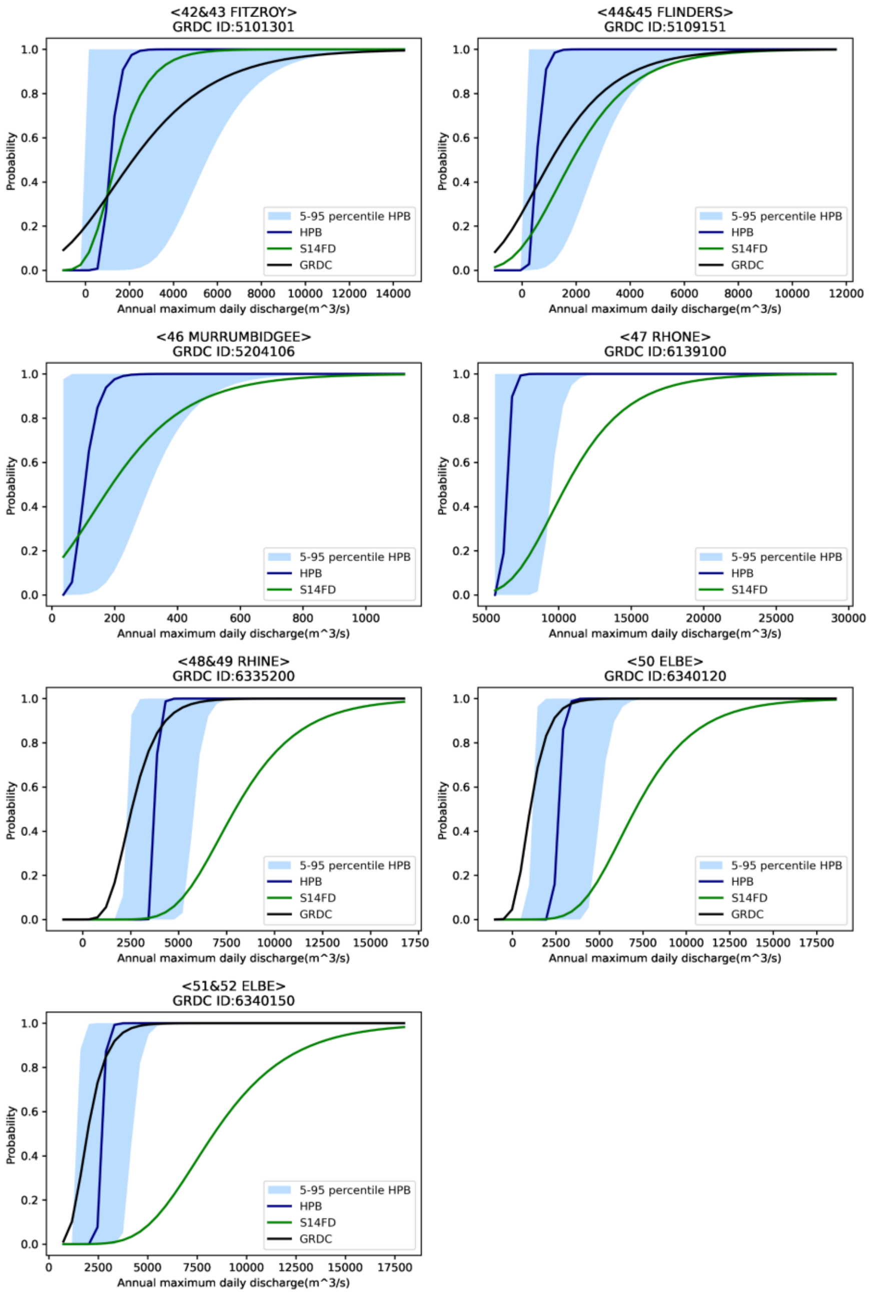

Figure S2. Comparison of cumulative density function (CDF) of annual maximum daily discharge from 1951–2010 for the GRDC, S14FD discharge reanalysis, and HPB (continuing...).

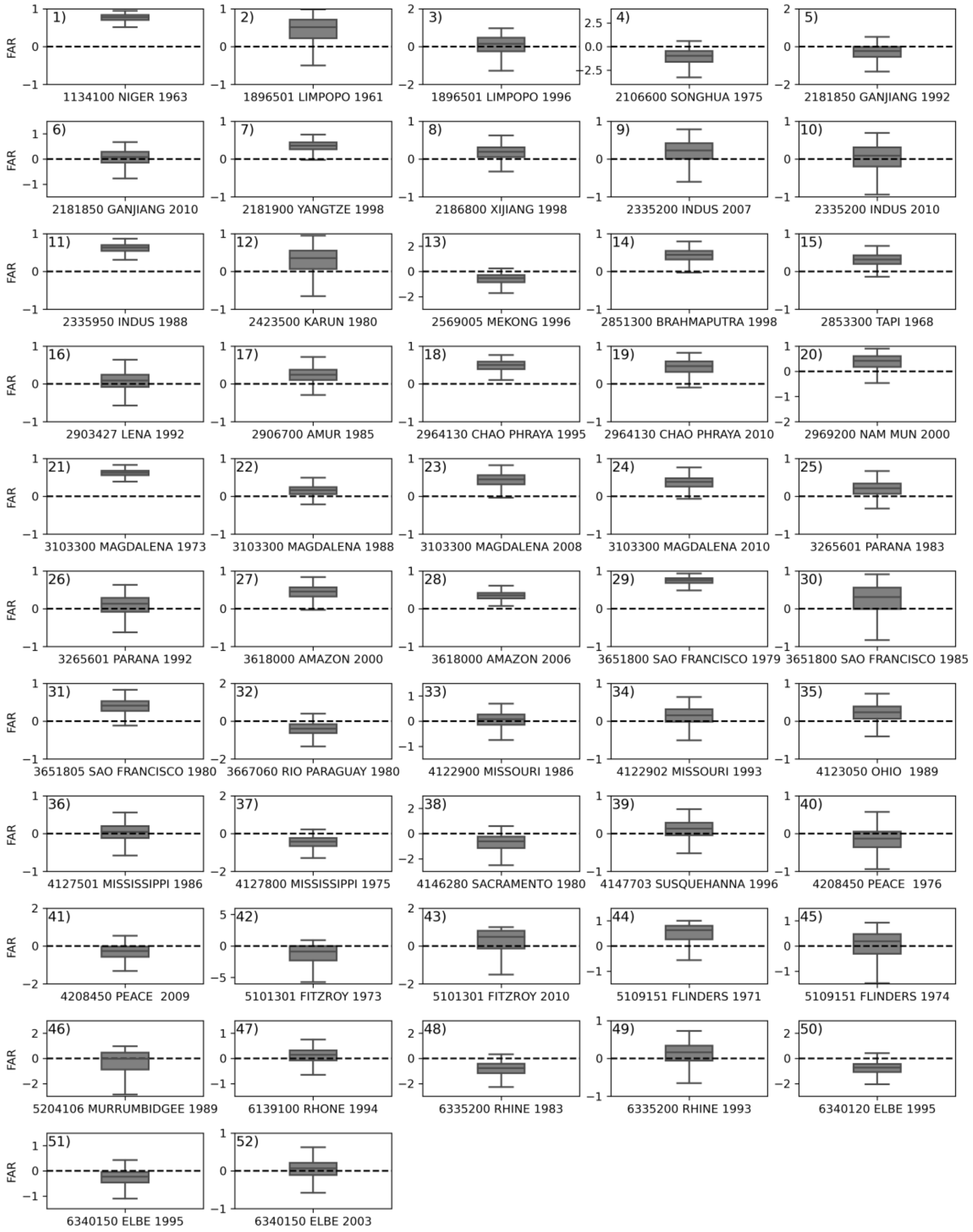

Figure S3. Uncertainty assessment of fraction of attributable risk (FAR) using the bootstrap technique.

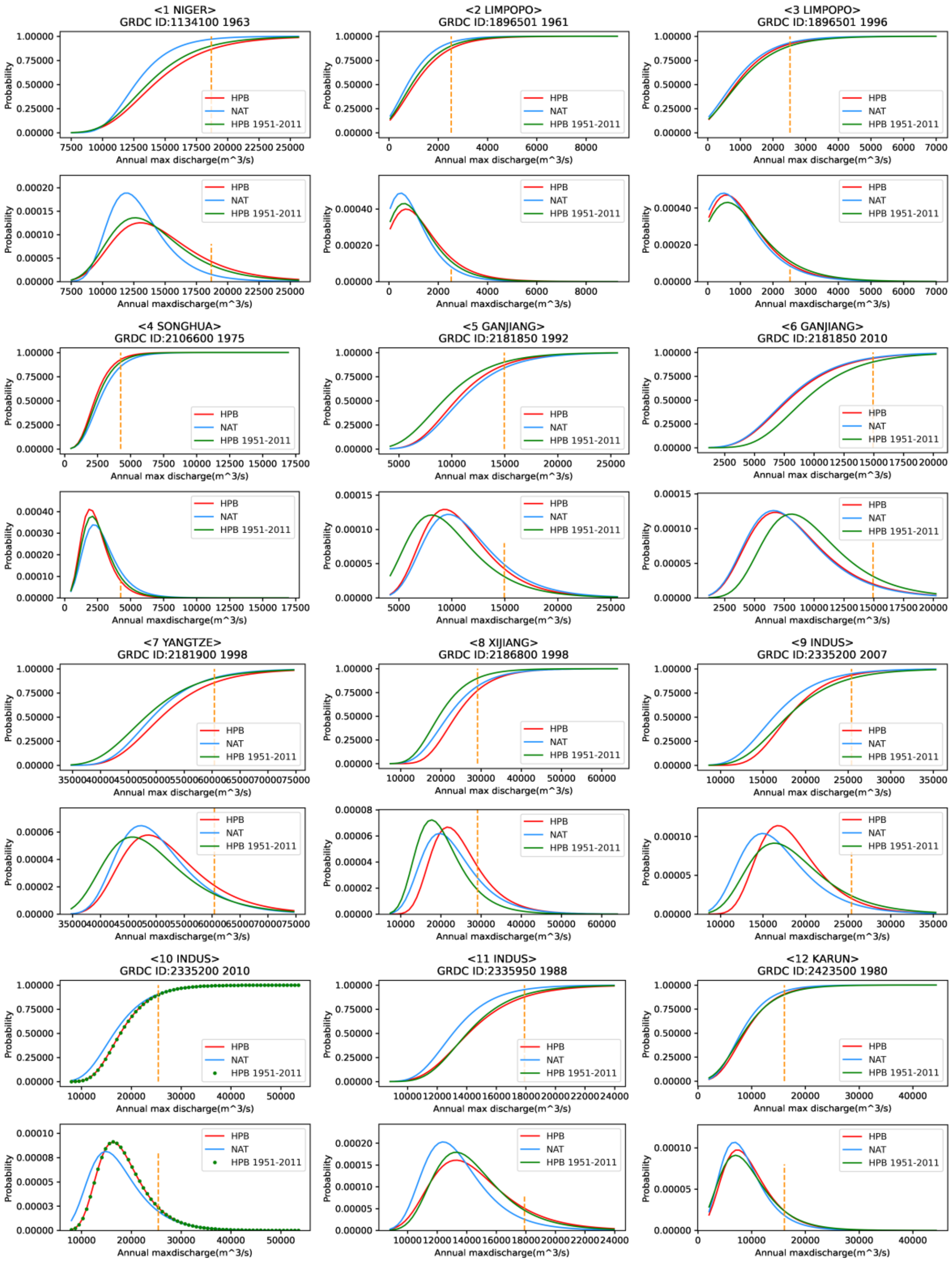

Figure S4. Comparison of cumulative and probability distribution function of annual maximum daily discharge (AMDD) of HPB and NAT experiments (Continuing…).

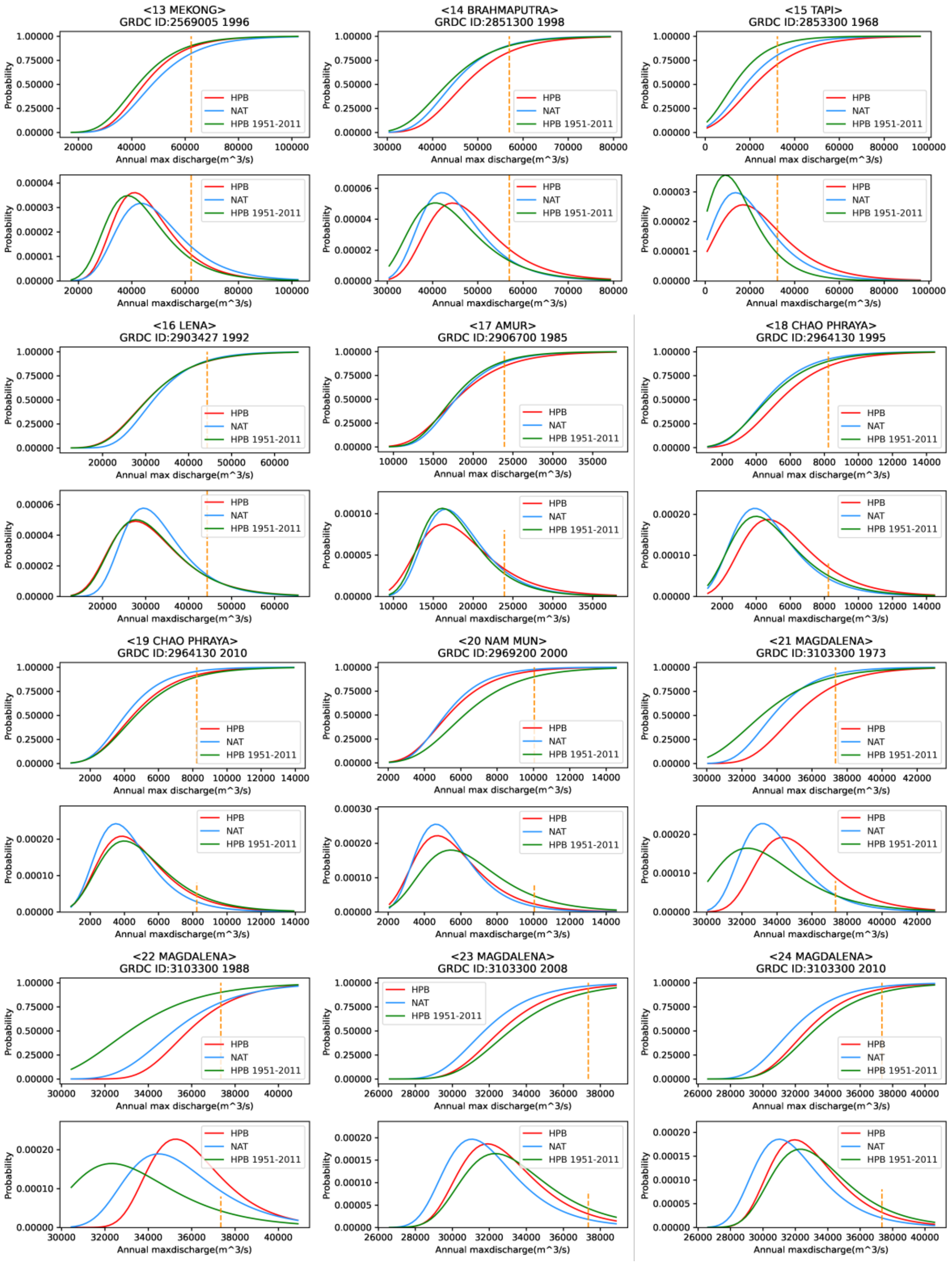

Figure S4. Comparison of cumulative and probability distribution function of annual maximum daily discharge (AMDD) of HPB and NAT experiments (Continuing…).

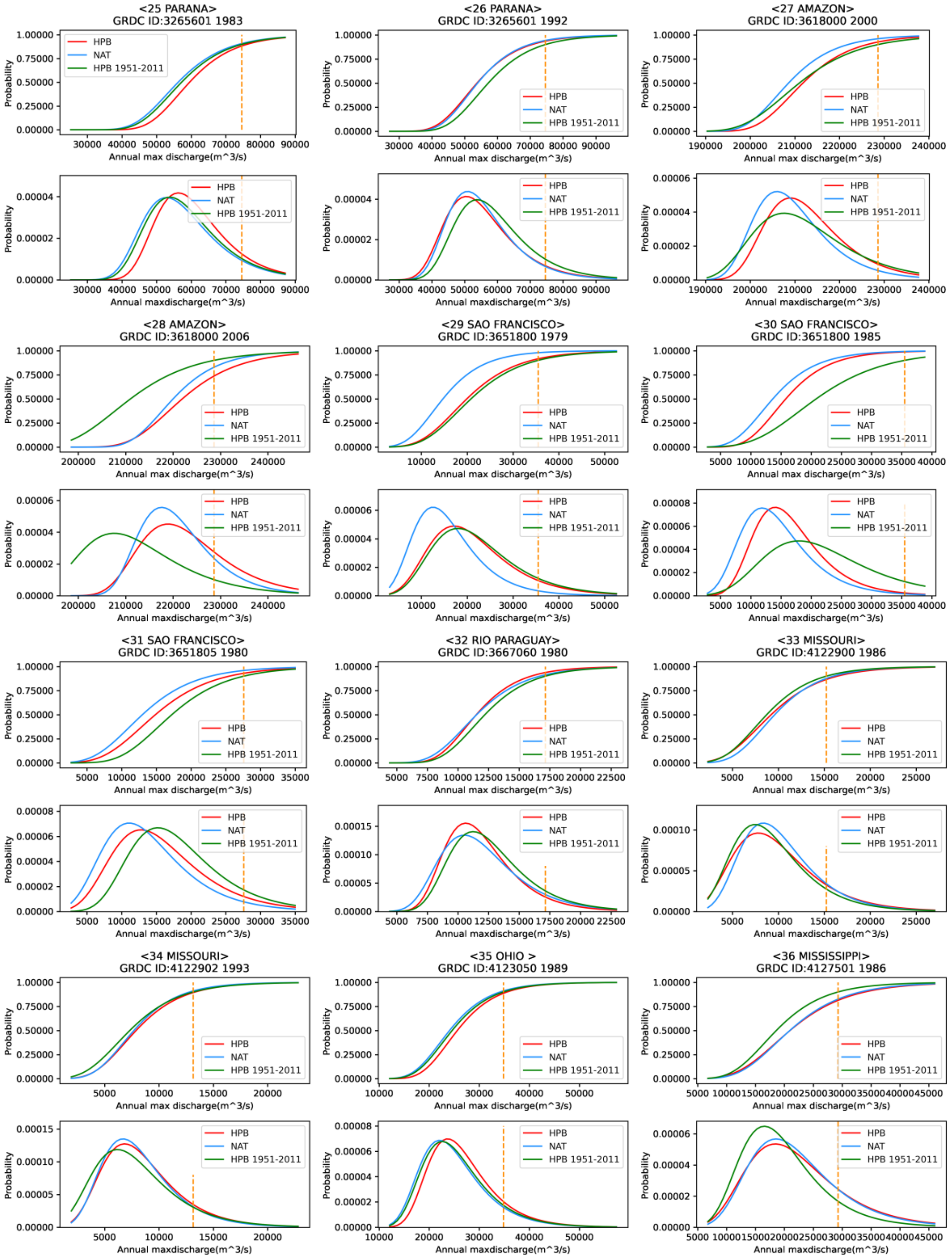

Figure S4. Comparison of cumulative and probability distribution function of annual maximum daily discharge (AMDD) of HPB and NAT experiments (Continuing…).

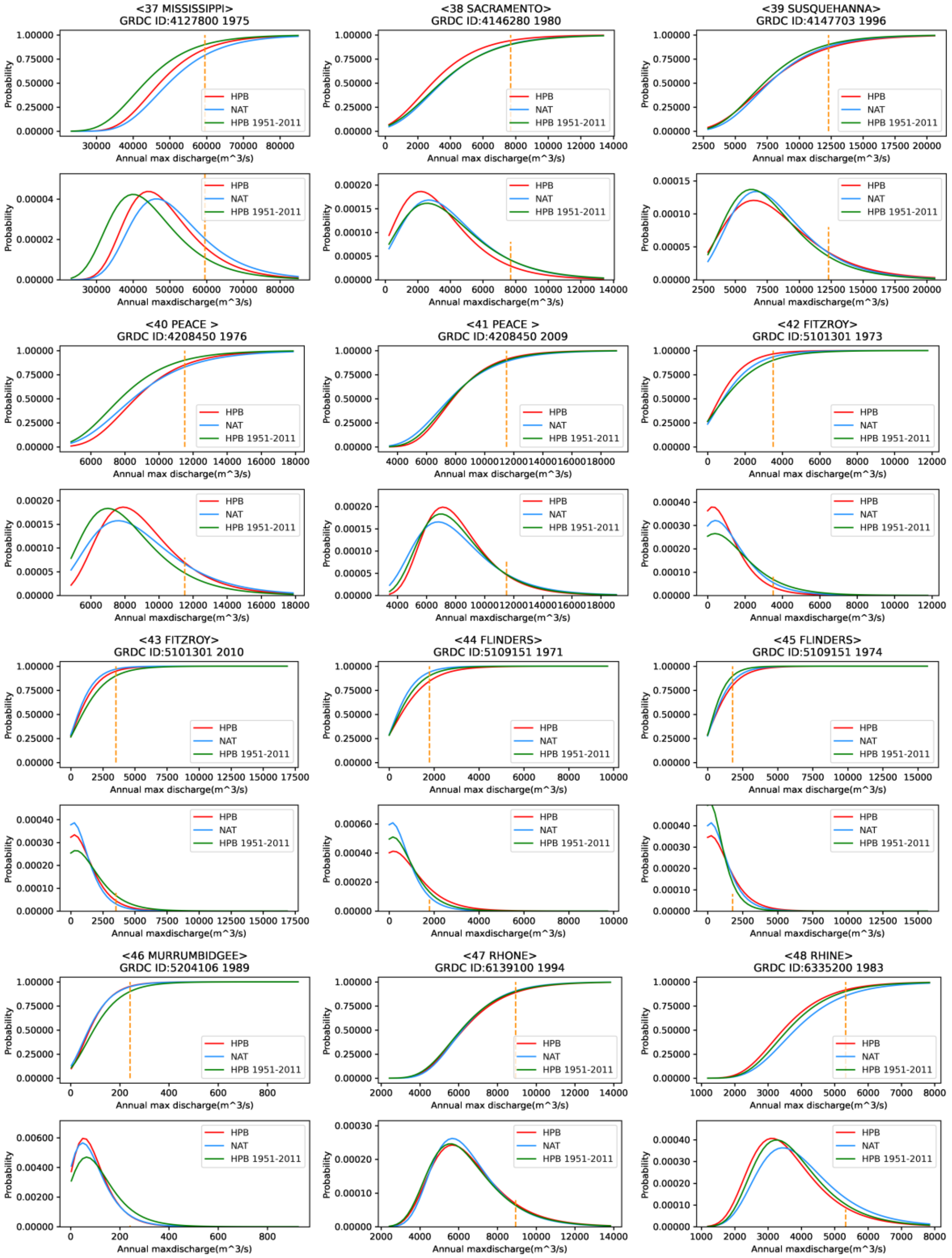

Figure S4. Comparison of cumulative and probability distribution function of annual maximum daily discharge (AMDD) of HPB and NAT experiments (Continuing…).

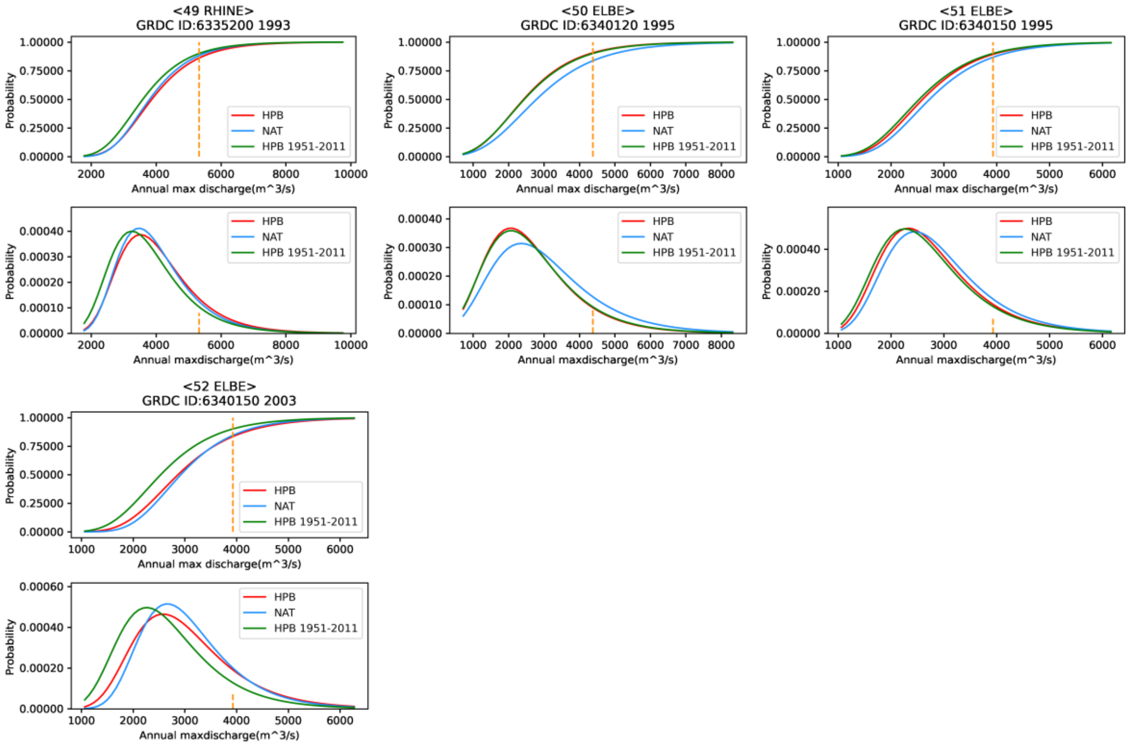

Figure S4. Comparison of cumulative and probability distribution function of annual maximum daily discharge (AMDD) of HPB and NAT experiments.

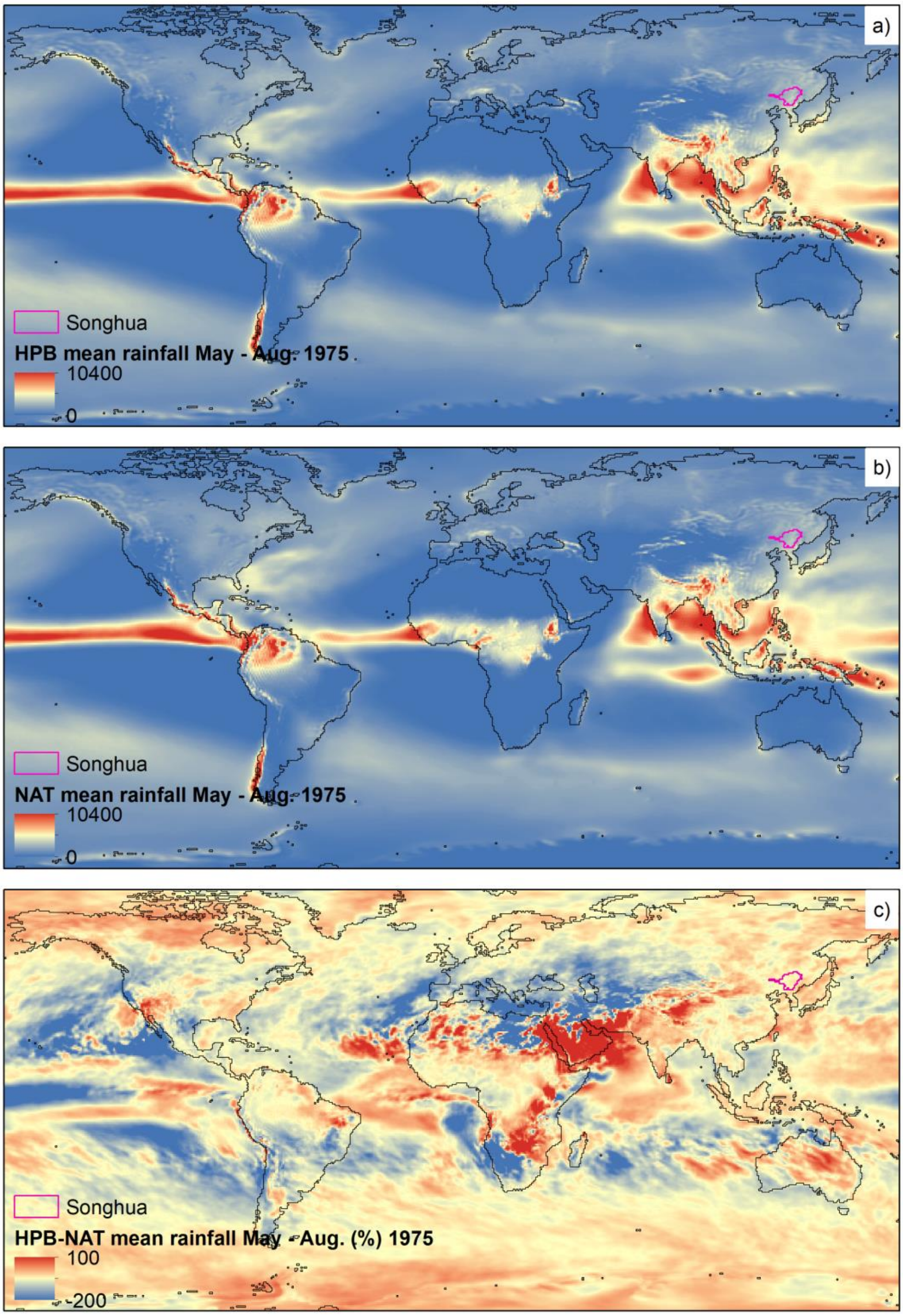

Figure S5. Mean rainfall from May- August from HPB (a), NAT (b), and percentage of difference between HPB and NAT (c). This figure was made using ESRI ArcGIS software (10.8.1).

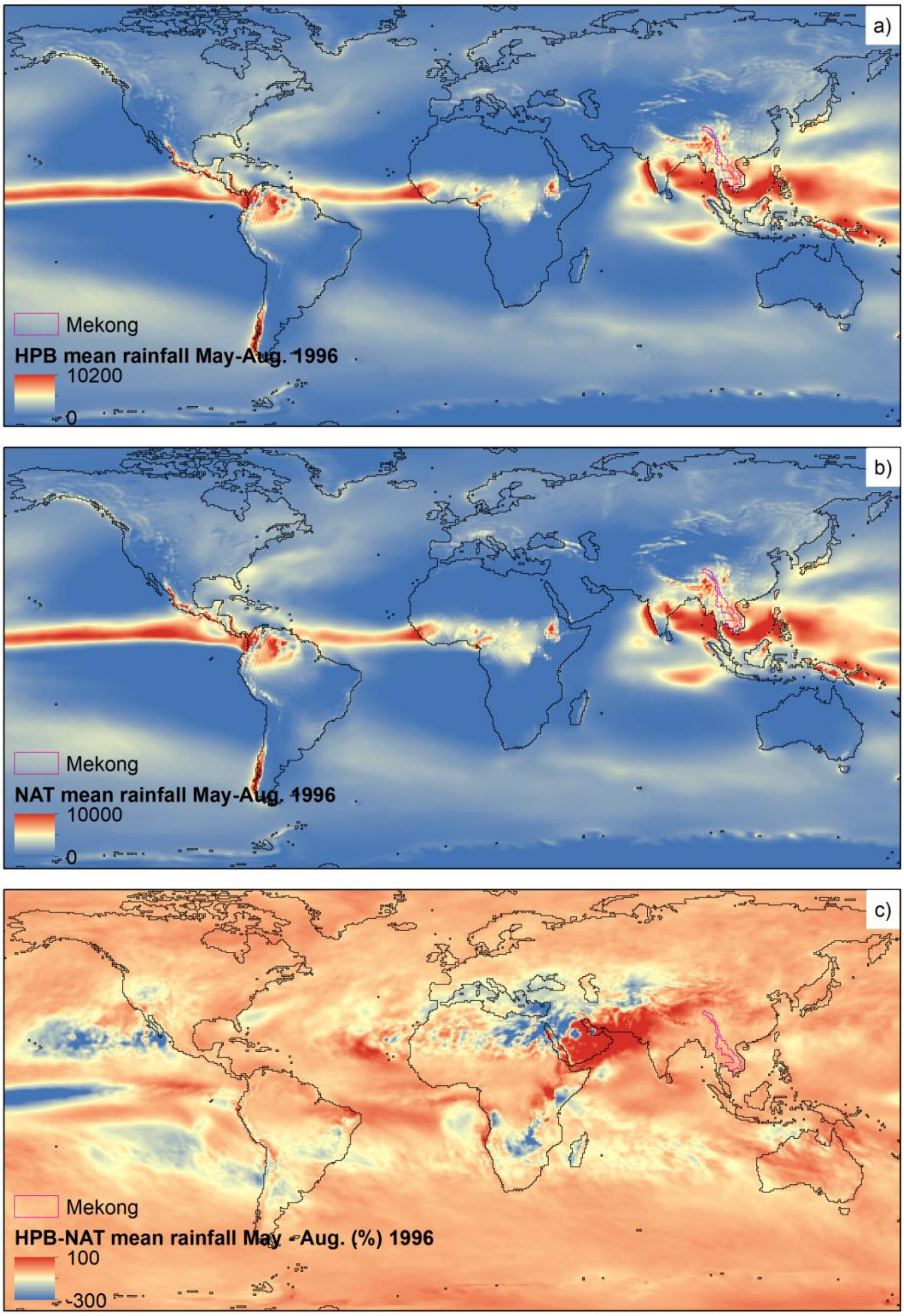

Figure S6. Mean rainfall from May- August from HPB (a), NAT (b), and percentage of difference between HPB and NAT (c). This figure was made using ESRI ArcGIS software (10.8.1).
